# Combined crystal structure prediction and high-pressure crystallization in rational pharmaceutical polymorph screening

**DOI:** 10.1038/ncomms8793

**Published:** 2015-07-22

**Authors:** M. A. Neumann, J. van de Streek, F. P. A. Fabbiani, P. Hidber, O. Grassmann

**Affiliations:** 1 Avant-garde Materials Simulation Deutschland GmbH, Merzhauser Strasse 177, D-79100 Freiburg, Germany; 2 Department of Pharmacy, University of Copenhagen, Universitetsparken 2, DK-2100 Copenhagen, Denmark; 3 Department of Crystallography, Georg-August-Universität Göttingen, GZG, Goldschmidtstrasse 1, D-37077 Göttingen, Germany; 4 F. Hoffmann-La Roche Ltd, Pharma Technical Development, Grenzacherstrasse 124, CH-4070 Basel, Switzerland; 5 Roche Pharmaceutical Research and Early Development, Roche Innovation Center Basel, F. Hoffmann-La Roche Ltd, Grenzacherstrasse 124, CH-4070 Basel, Switzerland

## Abstract

Organic molecules, such as pharmaceuticals, agro-chemicals and pigments, frequently form several crystal polymorphs with different physicochemical properties. Finding polymorphs has long been a purely experimental game of trial-and-error. Here we utilize *in silico* polymorph screening in combination with rationally planned crystallization experiments to study the polymorphism of the pharmaceutical compound Dalcetrapib, with 10 torsional degrees of freedom one of the most flexible molecules ever studied computationally. The experimental crystal polymorphs are found at the bottom of the calculated lattice energy landscape, and two predicted structures are identified as candidates for a missing, thermodynamically more stable polymorph. Pressure-dependent stability calculations suggested high pressure as a means to bring these polymorphs into existence. Subsequently, one of them could indeed be crystallized in the 0.02 to 0.50 GPa pressure range and was found to be metastable at ambient pressure, effectively derisking the appearance of a more stable polymorph during late-stage development of Dalcetrapib.

## Abstract (web summary)

Crystal polymorphism can lead to substances with vastly differing physicochemical properties, which has serious implications in the pharmaceutical industry. Here, the authors use *in silico* polymorph screening to accurately predict the resulting structures under set crystallisation environments.

## Graphical abstract

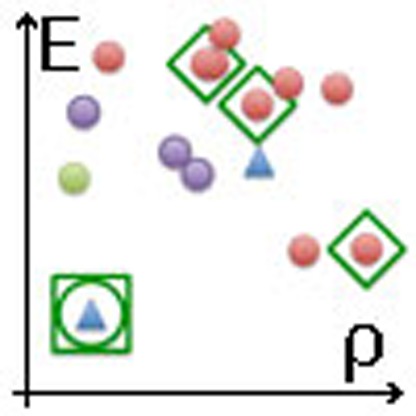

The ability of many chemical compounds to form different crystal structures is known as polymorphism^1^. The key physicochemical properties such as density, morphology, solubility, dissolution rate, electric conductivity, colour, birefringence, magnetic susceptibility and nonlinear optical properties are strongly influenced by the crystal packing. Polymorphism provides a chance for fine-tuning materials' properties, but also has important implications for manufacturing processes. For pharmaceuticals, polymorphism must be tightly controlled and is subjected to regulatory procedures, mainly because the crystal form can have a strong impact on the bioavailability of a drug and ultimately its therapeutic performance. In addition, novel polymorphs that show improved properties may be patented. A particular threat to the pharmaceutical industry is the phenomenon of ‘disappearing polymorphs'^2^. If the thermodynamically stable form has unfavourable crystallization kinetics, years may pass after initial synthesis before the first nucleation event; however, once the stable form has occurred it may prevent the production of the metastable form used in the drug formulation that is brought to market. A variation of this theme was experienced by Abbot^3^ in the Ritonavir case. Ritonavir was already marketed as a soft gel formulation, when an unexpected crystal form started to appear in 1998 with respect to which the chosen formulation turned out to be highly supersaturated. Subsequently, Ritonavir had to be reformulated at substantial cost and effort.

In response to the abovementioned risks and opportunities, experimental polymorph screening has become a standard procedure in pharmaceutical development. In numerous trial-and-error experiments carried out manually or with the help of robotic equipment, the key crystallization parameters such as solvent choice, antisolvent choice, evaporation rate and cooling rate are varied and the outcome is characterized by spectroscopic and thermoanalytical techniques, as well as diffraction measurements. However, there is no guarantee that a stable form with a high nucleation barrier will be obtained this way. The first nucleation event of such a form may require a special surface, the presence of an impurity or simply many repetitions of a certain crystallization experiment. The right conditions may not be encountered during the short time period of the experimental screening, but be matched accidentally after years of industrial manufacturing. An alternative approach would consist in mapping potentially stable polymorphs first by computational techniques, followed by a small number of well-targeted crystallization experiments to bring into existence potentially threatening crystal forms that have not yet been observed experimentally.

Tremendous progress has been achieved in the past decade in the field of crystal structure prediction (CSP) for both inorganic^4^,^5^,^6^,^7^,^8^,^9^ and organic compounds^10^,^11^,^12^. Both branches develop rather independently, because, despite many similarities, they involve different challenges and address different questions. For instance, in inorganic CSP the term ‘high pressure' will frequently refer to conditions found in a planet's interior, up to *ca.* 350 GPa at the centre of the Earth, or to conditions required to profoundly change the bonding and electronic properties of inorganic materials (for instance, sodium becomes transparent and insulating at *ca.* 200 GPa (ref. 8)), whereas in organic CSP it refers to a much narrower and more modest pressure range, typically 0.1–10 GPa, where most structural changes and phase transitions take place. Here we are exclusively concerned with CSP of organic compounds. The state-of-the-art in this field has been well documented by a series of blind tests^13^,^14^,^15^,^16^,^17^. Of the two main computational challenges, the complete sampling of the configurational space and the accuracy of the energy ranking, the first has to a large extent already been solved for structures crystallizing with one molecule per asymmetric unit. Energy ranking is still problematic when some low-energy structures are strongly stabilized by entropic contributions or when competing low-energy structures feature very different interactions such as dissimilar hydrogen-bonding schemes. However, the most important remaining challenge is probably the elaboration of clear crystallization recipes to cross the divide between the computational world (*in silico*) and the real world (*in vitro*). Since the 2010 blind test CSP studies of substantially flexible pharmaceutical molecules have started to appear in the scientific literature, with blind test compound XX as a model pharmaceutical^18^.

CSP has been shown to be a useful tool when it comes to rationalizing experimental observations involving pharmaceutical molecules, but further evidence needs to be built up to prove that it can deliver on its two most important industrial promises: lead experimentalists to new crystal forms and help to decide when it is safe to stop screening. In this article, we describe a real-life example of the pharmaceutical development compound Dalcetrapib, where both goals have been achieved to a large extent. A comprehensive CSP study for Dalcetrapib shows that in addition to the experimentally observed polymorphs, two predicted structures are candidates for a missing, thermodynamically more stable polymorph. The results of pressure-dependent stability calculations indicate that high-pressure conditions would favour the appearance of these high-density structures, thereby providing the recipe for a well-targeted crystallization experiment. Indeed, by following this recipe one of the two polymorphs can be crystallized in the 0.02 to 0.50 GPa pressure range. This crystal is metastable at ambient pressure, effectively derisking the appearance of a more stable polymorph during late-phase development of Dalcetrapib. This study illustrates how CSP and high-pressure crystallization can provide elements for rational pharmaceutical polymorph screening and in doing so bridge the divide between the computational and experimental worlds.

## Results

### Experimental polymorph screening at ambient pressure

**Figure 1:**
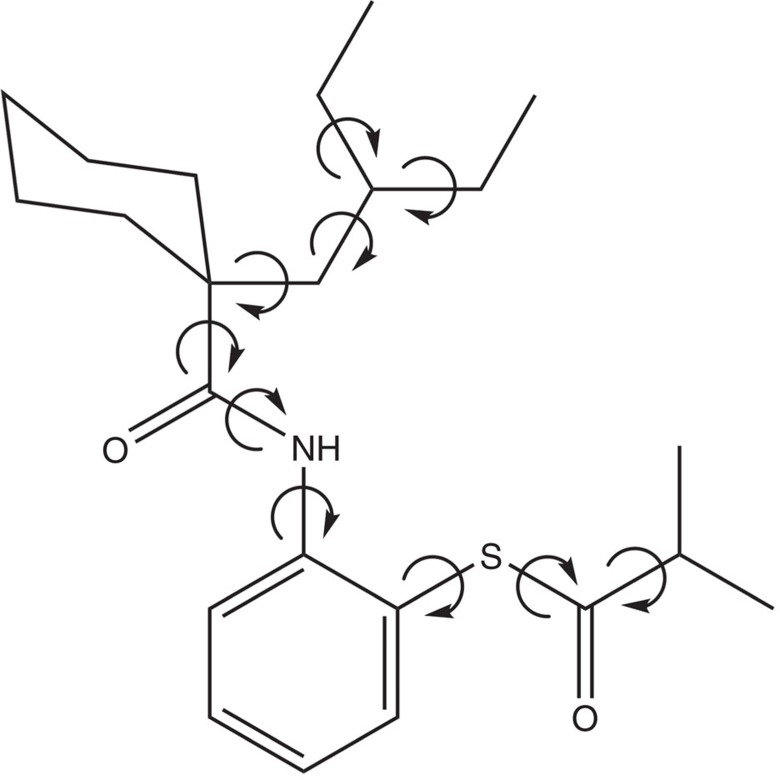
Molecular structure of Dalcetrapib. Chemical structure of Dalcetrapib highlighting the 10 torsional degrees of freedom.

Dalcetrapib^19^ (Fig. 1) was originally discovered by Japan Tobacco Inc., which reported in 2000 that the molecule inhibited cholesterol ester transferase protein activity. Dalcetrapib was in clinical development at Roche until 2012. It has been recently demonstrated that the drug compound has significant potential in treatment of cardiovascular diseases for a genetic subgroup^20^.

Extensive experimental crystal polymorph screens were performed to identify the relevant polymorphs of Dalcetrapib. In numerous solvent-based crystallization trials only one crystal polymorph, form A, was observed at ambient pressure and temperature. Differential scanning calorimetry and X-ray powder diffraction experiments reveal that at about −87 °C form A undergoes a reversible order–disorder phase transition to another polymorph, form B, that has been reported previously^21^ (see Supplementary Fig. 1). The crystal structures of forms A and B are almost identical, with the exception of the aliphatic side chains that are fully ordered in form B and highly disordered in form A (Supplementary Notes 1 and 2). The phase transition between forms A and B does not present a development risk because of its reversibility and the low phase-transition temperature.

### CSP of Dalcetrapib

The CSP method employed here, implemented in the computer programme GRACE, stood out in the last two blind tests^11^,^16^,^17^,^22^ in terms of success rate. It uses dispersion-corrected density functional theory (DFT-D) calculations^23^,^24^ for the final lattice energy ranking and for the generation of reference data to which a tailor-made force field^25^ is fitted. The tailor-made force field is used in a Monte-Carlo parallel-tempering algorithm with statistical convergence control to generate several thousand low-energy structures, which are then further processed at the DFT-D level. In terms of flexibility, the previous record in publicly available studies was held by blind test compound XX (ref. 17) with eight rotatable single bonds not counting terminal methyl groups. Here the limit is pushed further to 10 rotatable single bonds and an additional flexible six-membered ring for the pharmaceutical compound Dalcetrapib.

**Figure 2:**
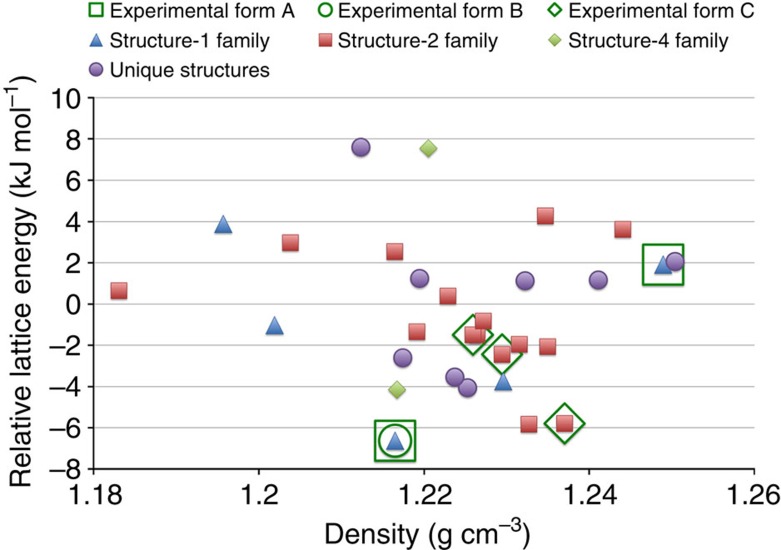
Energy-density diagram generated from the crystal structure prediction of Dalcetrapib. Energy-density diagram for the 30 computer-generated and three experimentally observed crystal structures of Dalcetrapib. Two of the experimental crystal forms are disordered and match several similar computer-generated structures. The computer-generated structures are numbered 1–30 in order of increasing lattice energy and families of similar structures are named according to their member with the lowest lattice energy. The average lattice energy was calibrated to zero.

An *in silico* polymorph screen with GRACE is conducted for Dalcetrapib in all 230 space groups with one molecule per asymmetric unit. Figure 2 shows an energy-density plot for the 30 most stable computer-generated crystal structures (see also Supplementary Table 1). Energy-density plots are commonly used as a graphical representation of the energy landscape. Each filled symbol corresponds to a local DFT-D lattice energy minimum. The computer-generated structures are numbered in the order of increasing lattice energy. Many of the structures are unique; however, there are also two large families of similar crystal structures. Within each family, the structures differ only with respect to the conformation of the alkyl chains. Structure 1 belongs to one of these families, and structure 2 to the other. The crystal structures in both families have *P*2_1_/*c* space-group symmetry, form almost identical unit cells and exhibit similar one-dimensional hydrogen-bonded chains; however, subsequent molecules in a chain are related by a twofold screw rotation for the structure 1 family and by a glide plane for the structure 2 family.

**Figure 3:**
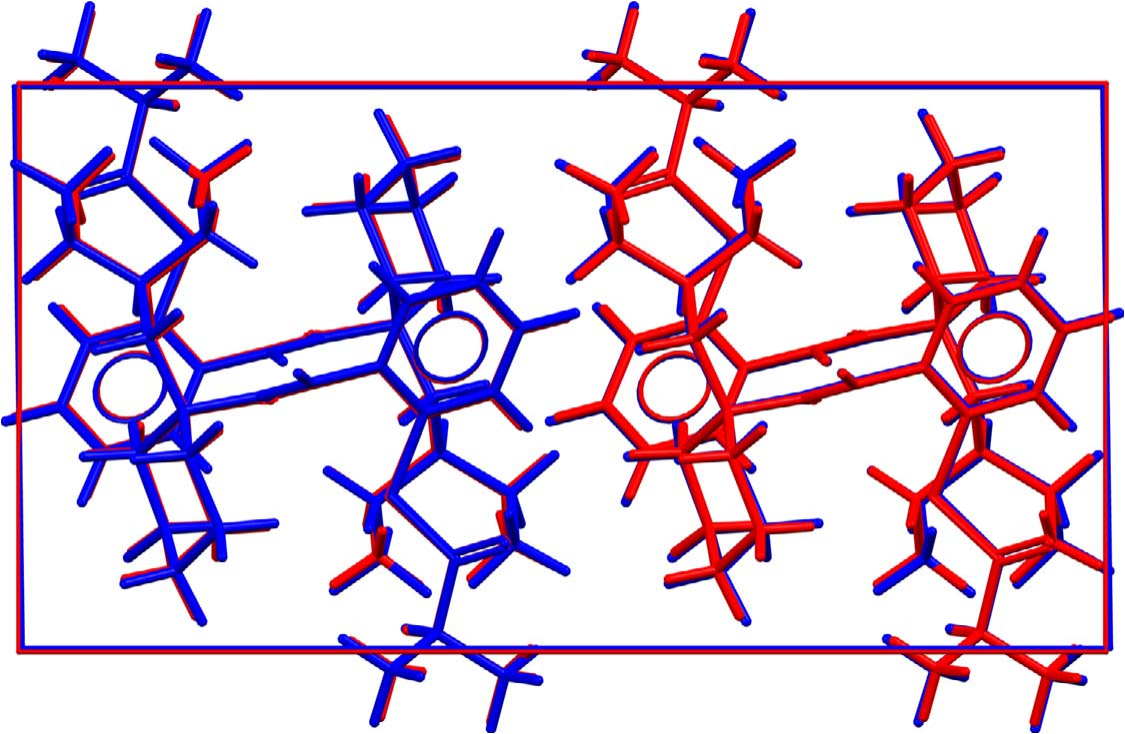
Structural overlay of experimental and computer-generated Dalcetrapib form B. Overlay of the experimental crystal structure of Dalcetrapib form B at 100 K (red) with the computer-generated crystal structure 1 (blue).

**Figure 4:**
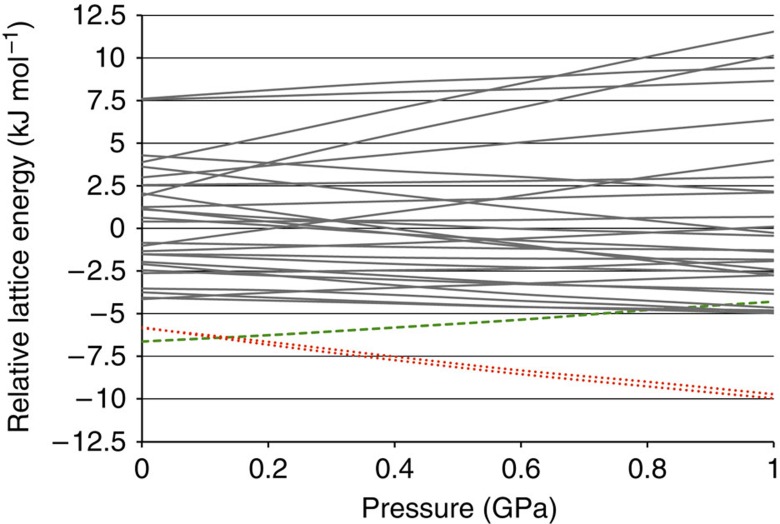
Relative lattice energies as a function of pressure. Relative lattice energies as a function of pressure for computer-generated structure 1 (green dashed line), structures 2 and 3 (red dotted lines) and all other computer-generated structures (continuous grey lines) of Dalcetrapib. Lattice energy optimizations were carried out from 0.0 to 1.0 GPa in steps of 0.2 GPa. For every pressure the average lattice energy was calibrated to zero.

The two experimental forms A and B both belong to the structure 1 family. The ordered low-temperature form B actually matches structure 1. Figure 3 shows an overlay of the two structures that is representative for the excellent agreement between experimental low-temperature structures and computer-generated structures at the DFT-D level. The atomic coordinates of the disordered high-temperature form A are compatible with a mixture of structure 1 and structure 22; however, lattice energy calculations presented in the Supplementary Note 1 suggest that the true nature of the disorder in form A is probably much more complex. The relative stability of crystal forms is determined by their free energies, to which the lattice energies reported here are only an approximation. In addition, various flavors of DFT-D yield somewhat different energy-ranking results. When Perdew-Burke-Ernzerhof (PBE)–Neumann–Perrin is used instead of BLYP-D3 (see Supplementary Note 1), structure 1 is predicted to be less stable than structures 2 and 3, which were therefore perceived as potential candidates for the true thermodynamically stable form. Pressure-dependent lattice energy calculations (see Fig. 4) indicate that at high pressure a form belonging to the structure 2 family would be significantly more stable than the experimentally observed forms A or B, thus providing a straightforward recipe for the crystallization of a new crystal form to be verified experimentally.

### Crystallization and stability of Dalcetrapib at high pressure

Indeed, such a form C was readily obtained by *in situ* high-pressure crystallization from solution^26^. The strength of this experimental technique for exploring the structural landscape of a compound at moderately high pressures (<1 GPa) is that any kinetic barrier associated with a molecular rearrangement in the solid state (as encountered in a polymorphic transformation by direct compression of the crystal) is bypassed by crystallization from solution directly under high-pressure conditions.

At conditions of ambient pressure, crystallization of Dalcetrapib is known to be kinetically hindered and solutions can be easily supersaturated. A 0.82 M tetrahydrofuran solution was loaded in the diamond-anvil cell (DAC) and crystallization of polycrystalline material was observed after *ca.* 24 h from initial loading, at a pressure of *ca.* 0.45 GPa. A single crystal suitable for single-crystal X-ray diffraction was grown by pressure and temperature cycling below *ca.* 0.1 GPa. Gentle heating (323–333 K) was applied to avoid compound decomposition. Stages of crystallization and crystal growth are depicted in Supplementary Fig. 3. During crystal growth the pressure inside the chamber drops significantly, indicating that the solid is considerably denser than the solution. The final pressure inside the chamber after crystal growth was *ca.* 0.02 GPa.

The structure solved from and refined against *in situ* single-crystal X-ray diffraction data matches the computer-generated structure 3 (Supplementary Figs 4–6 and Supplementary Table 2), which is the second lowest-energy structure in the structure 2 family. The experimentally observed disorder of the aliphatic side chains is consistent with a model of thermally populated molecular conformations, taken from structures 9 and 13 (and to a lesser extent 2 and 12, see Supplementary Tables 2–3 and Supplementary Fig. 6) of the structure 2 family, in the ordered but relaxed crystalline environment of structure 3. Lattice energy calculations for various disorder models show that structure 2 is less affected by thermodynamic disorder than structure 3. Structure 3 is observed experimentally instead of structure 2 because of the contribution of disorder to the free energy.

For some chemical compounds the crystal forms obtained at high pressure are sufficiently stable to be recovered at ambient pressure, as testified by the numerous examples of novel inorganic materials obtained through high-pressure synthesis^27^,^28^,^29^. In the case of molecular organic materials, recovered crystals can additionally be used as seeds for further crystallization experiments. This was recently demonstrated for the analgesic acetaminophen^30^ and the neurotransmitter γ-aminobutyric acid^31^. Successful recovery and seeding experiments are of particular interest for industrial applications, especially when high pressure is the sole or most reliable route for obtaining a particular solid form, as is the case for Dalcetrapib form C. Therefore, several attempts were made to recover form C at ambient conditions (see Supplementary Note 2 and Supplementary Figs 7–11 for full details). When crystallized from solution in the 0.02 to 0.50 GPa pressure range, form C instantly converts to form A in a solution-mediated transformation on opening of the diamond anvil cell. When form C is crystallized from the melt at high pressure and the pressure is released without opening the diamond anvil cell, form C can be obtained at ambient pressure but converts to form A on a timescale of hours. Both experiments prove that form A is more stable than form C at ambient conditions.

### Rational crystallization experiments from energy landscapes

Abramov^32^ provided an assessment of the usefulness of CSP to support solid-form selection. The energy landscape of the still rather rigid pharmaceutical olanzapine was used by Bhardwaj *et al.*^33^ to rationalize some of the observed crystallization behaviour, including the low packing efficiency of the unsolvated forms and the role of solvent in stabilizing the solvate structures. Two out of three experimental unsolvated polymorphs were identified among the computer-generated crystal structures. Four structures were predicted to be more stable than all of the experimental polymorphs; however, the crystallization of a new polymorph using knowledge of the energy landscape was not reported. Ismail *et al.*^34^ were successful in finding the only known polymorph of a compound named GSK269984B, previously in development at GlaxoSmithKline, as the most stable polymorph in their prediction. GSK269984B is only slightly less flexible than the compound studied here. The energy landscape revealed many crystal packings with lattice energies close to the experimentally observed one, featuring a variety of molecular conformations. On the basis of this knowledge new crystallization experiments were designed to generate new crystal forms; however, no additional polymorphs could be observed experimentally. The authors noted that unlike the known polymorph many computer-generated structures did not match the most stable conformation of the molecule computed in isolation. The inability to obtain new polymorphs was rationalized in terms of expected poor crystallization dynamics because of the necessity for conformational change during the crystallization process. The aforementioned studies and the work presented here have in common that in the relevant energy window for polymorphism there are far more computer-generated structures than observed polymorphs. Price^35^ has proposed a comprehensive analysis of this observation.

It is not new that additional crystal polymorphs of organic molecules may be obtained by high-pressure crystallization, or that high-pressure forms are likely to be found in CSP studies to have higher densities than ambient-pressure forms. In a collaborative effort between experimentalists and computational scientists carried out under blind test conditions, Oswald *et al.*^36^ experimentally obtained both a low-density form at ambient pressure and a high-density form at high pressure for each of the molecules 2-chlorophenol and 4-fluorophenol. All experimental polymorphs were found in the CSP studies with the correct density relationships. However, the CSP studies did actually not correctly predict the appearance of the high-density polymorphs at high pressure because in both cases many computer-generated structures were computed to be more stable than the experimentally observed ones at the crystallization pressure. For 2-chorophenol, the calculations did not show a significant stabilization with pressure of the high-density polymorph in comparison with the low-density polymorph. In another case, Day *et al.*^37^ obtained and characterized a previously unknown polymorph, form II, of maleic acid serendipitously by dissolution of the 1:2 cocrystal between maleic acid and caffeine in chloroform, but were unsuccessful to obtain the new polymorph a second time. In a subsequent CSP study published with the experimental work, the authors found both polymorphs correctly among the most stable computer-generated structures, with form II being predicted to have higher density in agreement with experimental findings. Later, Oswald *et al.*^30^ demonstrated that form II can be reproducibly obtained by high-pressure crystallization. While highly correct, the CSP study on maleic acid provides a postdiction, rather than a prediction of a high-density polymorph obtained at high pressure. 2-chorophenol, 4-fluorophenol and maleic acid are all very small molecules by pharmaceutical standards. One novel aspect of our work is to demonstrate that even for highly flexible pharmaceutical molecules, crystal energy landscapes and their pressure dependence can be calculated with high-enough accuracy to convince industrial crystallization scientists to deviate from their experimental standard protocols and invest additional resources to obtain a specific predicted crystal structure experimentally, through a high-pressure crystallization experiment at a pressure suggested by theory.

With the structure 2 family having been observed experimentally at high pressure, our computational results for Dalcetrapib show no indication for having missed the thermodynamically stable form at ambient conditions. Within each family, cross-nucleation and low-energy barriers for solid–solid phase transitions make it unlikely to encounter a metastable form that does not readily convert to the most stable form in the family; therefore, none of the structures in the two observed families presents a danger regardless of potential computational errors. The structure 4 family and structure 5 exhibit the same hydrogen bonding and a similar molecular conformation as the experimental structures. Hence, there is in principle no apparent reason why their nucleation or growth should be hindered compared with the observed forms. If any of them were the truly stable form, they should have been observed in the experimental screening experiments, whereas only forms A and B, which are thermodynamically the two most stable forms, were repeatedly isolated. Interestingly, this argument also applies to form C; however, this low-energy form could be isolated experimentally by changing another thermodynamic variable, namely pressure, in the crystallization experiments, and effectively moving to and probing another portion of the compound's phase diagram. Once more experience with interpreting crystal energy landscapes has been built up, it may well turn out that form C should never have been considered a candidate for a missing thermodynamically stable form at ambient conditions. For an unobserved low-energy structure to be flagged as a threat after thorough experimental screening, it should probably also present a structural feature suggesting a high-nucleation barrier, such as an unlikely molecular conformation according to the statistics of the Cambridge Structural Database. Some of the unique structures feature other molecular conformations; however, they are all predicted to be less stable than structure 1 by more than 2 kJ mol^−1^ and cannot benefit from a reduction in the configurational free energy by disorder because they are not part of a family. For Dalcetrapib the calculated lattice energy ranking yields a reliable description of the relative stability because of the strong structural similarity between the computer-generated low-energy structures.

## Discussion

The reader may wonder why high-pressure crystallization should not simply be added to the experimental trial-and-error screening protocols without the need to computationally generate a crystal energy landscape. While there are signs that the rules of the game are slowly changing and high-pressure experiments on pharmaceuticals are attracting some industrial interest in their own right, the answer still is that it is always easier to find something when you know what you are looking for. High-pressure experiments need the intervention of an academic specialist, are time-consuming and usually require diamond anvil cells as crystallization vessels, limiting the choice of crystallization conditions and capability of parallel screening compared with crystallization at ambient conditions. In the case of Dalcetrapib, initial experiments gave no indication of crystallization from the melt under pressure 1 month after having initially loaded the diamond anvil cell (see Supplementary Note 2). It would be unlikely that a crystallization experiment had continued for such a long time in an industrial context without knowing that there was something to look for. High-pressure crystallization from solution of form C occurred after only a day; however, even in this case no crystallization event might have been observed in an industrial laboratory without prior, time-consuming optimization of high-pressure crystallization conditions. In the case that another high-density form had crystallized first and repeatedly so, an expensive high-pressure screen might have been stopped after a few attempts. The computed crystal energy landscape is not perfect; however, it roughly tells the experimentalist in which pressure range new forms can be expected, how stable these forms will be compared with other known or predicted structures and whether they are stable enough at ambient conditions to be likely candidates for the thermodynamically most stable form. The energy landscape may also help to get the crystallization conditions right. If the molecular conformation in a predicted high-density form matches a known form, the high-pressure form is likely to crystallize from solvents from which the known form could be obtained. Of course, the crystallization recipe obtained from computer simulations is still limited. Which form, if any, results from a specific high-pressure crystallization experiment may depend on subtle conditions such as the rate of compression^38^,^39^ and ultimately remains a question of trial-and-error, even though the likelihood of success is high. In addition, only an experimental investigation will show if a new form can be recovered at ambient conditions. If so, further investigations will typically be required to find other crystallization conditions under which the new form can be obtained reproducibly and in large quantities.

The art of bringing into existence predicted crystal structures for physical characterization is only in its infancy. A toolbox of specific techniques will have to be developed that can be used to crystallize virtually any predicted structure not too far away from the global energy minimum. In that toolbox, crystallization under pressure will be the method of choice for high-density structures. The knowledge of the predicted crystal structures may help to choose solvents that favour certain molecular conformations or associations^40^. Another versatile route that offers much promise is the use of tailor-made additives^41^. Removing hydrogen-bond acceptors from the molecule to be crystallized or replacing hydrogen atoms with halogen atoms, it should be possible to design additives that are easily incorporated in a candidate form, but do not fit into the experimentally observed forms and thereby act as crystal growth inhibitors^42^. Or why not search computationally among thousands of commercially available compounds with known crystal structures for a crystal surface that would be the ideal substrate for the heterogeneous nucleation of a candidate form^43^? Crystallization in electric fields^44^, ultrasound fields^45^ or laser beams^46^ provides further avenues for exploration, provided that the effect of these experimental conditions on the growth of specific crystal structures is understood.

## Methods

### General

Detailed information on the computational, experimental and crystallographic methods is provided in the Supplementary Notes 1 and 2, Supplementary Figs 12–15 and Supplementary Table 4.

### Energy calculations

All DFT-D lattice energy optimizations have been carried out with the GRACE programme. For the calculation of DFT energies and forces, GRACE calls the *ab initio* total-energy and molecular-dynamics programme Vienna Ab initio Simulation Package (VASP) developed at the ‘Institut für Materialphysik' of Vienna University^47^,^48^,^49^,^50^. The dispersion correction is implemented in GRACE. The energy calculation method BLYP-D3 combines the BLYP functional, implemented in VASP, with the dispersion correction according to ref. 24, implemented in GRACE. The energy method PBE–Neumann–Perrin combines the PBE functional with the dispersion correction according to ref. 23. DFT calculations use a plane wave cutoff energy of 520 eV and a k-point spacing of roughly 0.07 Å^−1^. All lattice energy minimizations have been converged to within at least 0.003 Å for atomic displacements, 0.001 kJ mol^−1^ per atom for energy changes, 1.7 kJ mol^−1^ per Å for atomic forces and 0.0125, kbar for cell stress. The lattice energies of the 30 most stable computer-generated crystal structures according to BLYP-D3 are listed in Supplementary Table 1 both for BLYP-D3 and PBE–Neumann–Perrin. On average, the lattice energies calculated with both methods deviate from each other by 1.9 kJ mol^−1^ per molecule. The 30 structures resulting from the BLYP-D3 optimization are provided as Supplementary Data. Considering the possibility of disorder significantly complicates the tasks of CSP. Therefore, the blind test compounds have always been selected as to avoid disordered structures and typically compounds with ordered crystal structures are chosen for CSP validation studies. However, disorder is a commonly observed phenomenon in molecular crystals and can have a strong impact on the relative stability of crystal forms because of its contribution to the crystal free energy. Modelling and rationalization of structural disorder has been shown to be amenable to CSP, as demonstrated in three recent examples on pharmaceuticals^51^,^52^,^53^. With two disordered crystal polymorphs out of three, Dalcetrapib exhibits a particularly high degree of disorder and the energy ranking presented here would have been incomplete without an attempt to quantify the configurational contribution to the crystal free energy by means of lattice energy calculations. Full information on the treatment of disorder in the calculations is detailed in the Supporting Note 1 and Supporting Fig. 2. A rigorous treatment of disorder as applied to caffeine^54^ is still out of scope at the DFT-D level for a molecule such as Dalcetrapib because of the high CPU time requirements.

The field of dispersion-inclusive DFT is rapidly evolving, and it is appropriate to mention some recent developments that were not used in the present work. Various ways of treating van der Waals dispersion forces in DFT have recently been classified according to ref. 55. A particularly promising approach that combines DFT with multibody dispersion interactions was shown to reproduce the lattice enthalpy differences between the polymorphs of glycine and oxalic acid with an accuracy of ∼1 kJ mol^−1^ (ref. 56). For the assessment of these and other methods, a benchmark for non-covalent interactions in solids derived from sublimation enthalpies is now available^57^. Quantum mechanics-based fragment methods provide a systematically improvable approach to make predictions in the condensed phase for the crystal structures of small organic molecules^58^ and may become a reliable source of theoretical benchmark data.

### High-pressure experiments

A modified Merrill–Bassett DAC^59^ equipped with 800-μm culet diamonds mounted on WC seats and Inconel steel gaskets of 300-μm diameter was used for all experiments. The opening angle of the cell for X-rays was 84°. Single-crystal diffraction data were collected at 295 K on a Bruker Apex II diffractometer equipped with Mo-sealed tube radiation. A standard data collection strategy that aims at optimizing data redundancy and completeness was used. Recovery experiments were performed on single-crystal and polycrystalline samples of form C and the obtained material analysed using X-ray diffraction. Single crystals were grown *in situ* from solution in the DAC; polycrystalline samples were grown *in situ* either from solution or from the melt in the DAC.

## Additional information

**Accession codes:** Crystallographic data deposition. The X-ray crystallographic coordinates for structures reported in this study, C (295 K and 0.02 GPa), A (295 and 223 K) and B (100 K) have been deposited at the Cambridge Crystallographic Data Centre (CCDC), under deposition numbers 1024144–1024147. These data can be obtained free of charge from The Cambridge Crystallographic Data Centre via www.ccdc.cam.ac.uk/data_request/cif.

**How to cite this article**: Neumann, M. A. *et al.* Combined crystal structure prediction and high-pressure crystallization in rational pharmaceutical polymorph screening. *Nat. Commun.* 6:7793 doi: 10.1038/ncomms8793 (2015).

## Supplementary Material

Supplementary Figures, Supplementary Tables, Supplementary Notes and Supplementary References Supplementary Figures 1-15, Supplementary Tables 1-4, Supplementary Notes 1-2 and Supplementary References

Supplementary Data 1 CIF file for Form C at 0.02 GPa

Supplementary Data 2 CIF file for Form A at 295 K

Supplementary Data 3 CIF file for Form A at 223 K

Supplementary Data 4 CIF file for Form B at 100 K

Supplementary Data 5 Predicted structure 1

Supplementary Data 6 Predicted structure 2

Supplementary Data 7 Predicted structure 3

Supplementary Data 8 Predicted structure 4

Supplementary Data 9 Predicted structure 5

Supplementary Data 10 Predicted structure 6

Supplementary Data 11 Predicted structure 7

Supplementary Data 12 Predicted structure 8

Supplementary Data 13 Predicted structure 9

Supplementary Data 14 Predicted structure 10

Supplementary Data 15 Predicted structure 11

Supplementary Data 16 Predicted structure 12

Supplementary Data 17 Predicted structure 13

Supplementary Data 18 Predicted structure 14

Supplementary Data 19 Predicted structure 15

Supplementary Data 20 Predicted structure 16

Supplementary Data 21 Predicted structure 17

Supplementary Data 22 Predicted structure 18

Supplementary Data 23 Predicted structure 19

Supplementary Data 24 Predicted structure 20

Supplementary Data 25 Predicted structure 21

Supplementary Data 26 Predicted structure 22

Supplementary Data 27 Predicted structure 23

Supplementary Data 28 Predicted structure 24

Supplementary Data 29 Predicted structure 25

Supplementary Data 30 Predicted structure 26

Supplementary Data 31 Predicted structure 27

Supplementary Data 32 Predicted structure 28

Supplementary Data 33 Predicted structure 29

Supplementary Data 34 Predicted structure 30

Supplementary Data 35 Structure 1 configuration 1 (see Supplementary Table 2)

Supplementary Data 36 Structure 1 configuration 2 (see Supplementary Table 2)

Supplementary Data 37 Structure 2 configuration 1 (see Supplementary Table 2)

Supplementary Data 38 Structure 2 configuration 2 (see Supplementary Table 2)

Supplementary Data 39 Structure 2 configuration 3 (see Supplementary Table 2)

Supplementary Data 40 Structure 2 configuration 4 (see Supplementary Table 2)

Supplementary Data 41 Structure 3 configuration 1 (see Supplementary Table 2)

Supplementary Data 42 Structure 3 configuration 2 (see Supplementary Table 2)

Supplementary Data 43 Structure 3 configuration 3 (see Supplementary Table 2)

Supplementary Data 44 Structure 3 configuration 4 (see Supplementary Table 2)

Supplementary Data 45 Structure 3 configuration 5 (see Supplementary Table 2)

Supplementary Data 46 Form A hypothesis configuration 1 (see Supplementary Table 3)

Supplementary Data 47 Form A hypothesis configuration 2 (see Supplementary Table 3)

Supplementary Data 48 Form A hypothesis configuration 3 (see Supplementary Table 3)

Supplementary Data 49 Form A hypothesis configuration 4 (see Supplementary Table 3)

Supplementary Data 50 Form A hypothesis configuration 5 (see Supplementary Table 3)

Supplementary Data 51 Form A hypothesis configuration 6 (see Supplementary Table 3)
