# Supplementary material for: Combined crystal structure prediction and high-pressure crystallization in rational pharmaceutical polymorph screening
Source: Nat Commun. 2015 Jul 22;6:7793. doi: 10.1038/ncomms8793 (PMC4525153; doi:10.1038/ncomms8793)
Supplement: Supplementary Figures, Supplementary Tables, Supplementary Notes and Supplementary References — Supplementary Figures 1-15, Supplementary Tables 1-4, Supplementary Notes 1-2 and Supplementary References [file ncomms8793-s1.pdf]

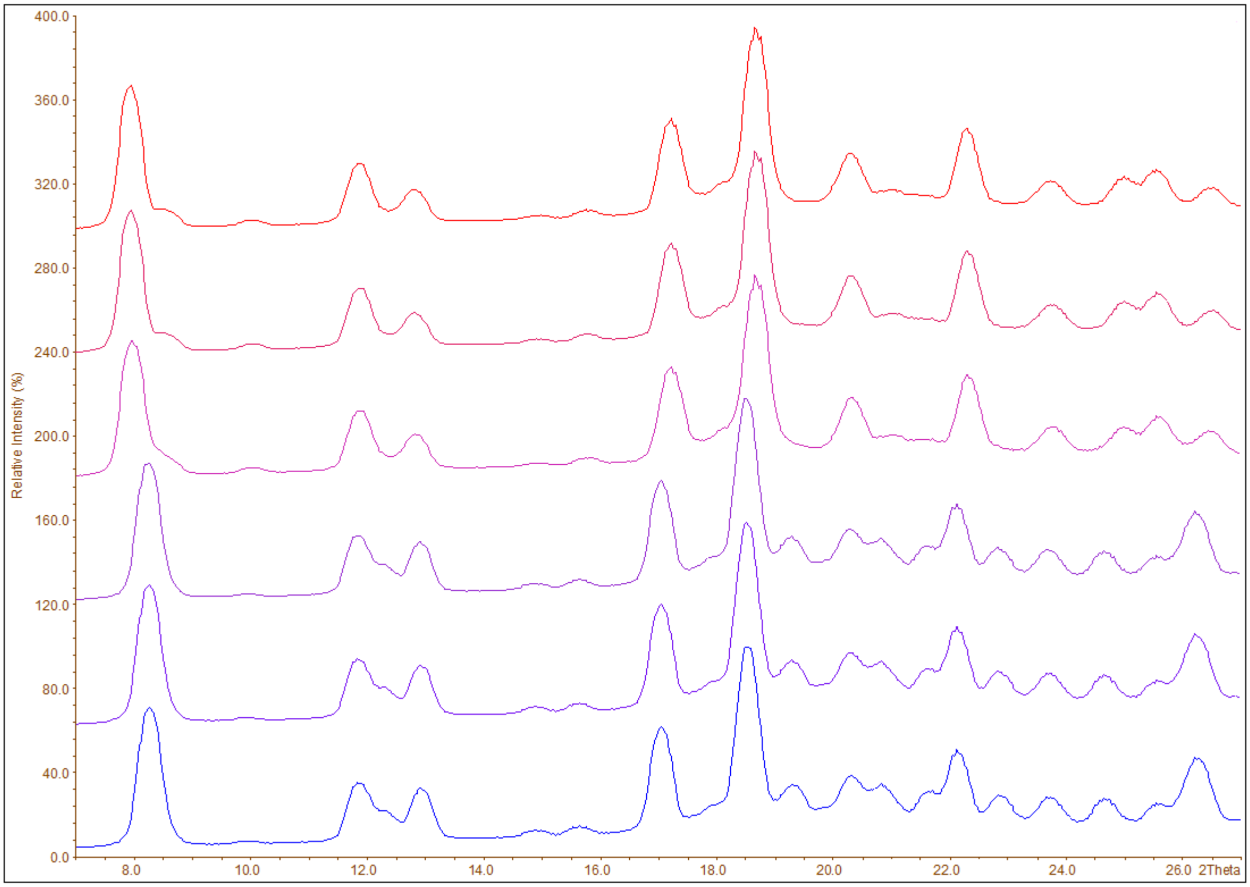

Supplementary Figure 1. Experimental powder patterns of forms B and A. The X-ray diffraction patterns were recorded at -110, -100, -90, -80, -70, -60°C (bottom to top). The patterns recorded at -110, -100, -90°C correspond to form B, patterns recorded at -80, -70, -60°C correspond to form A.

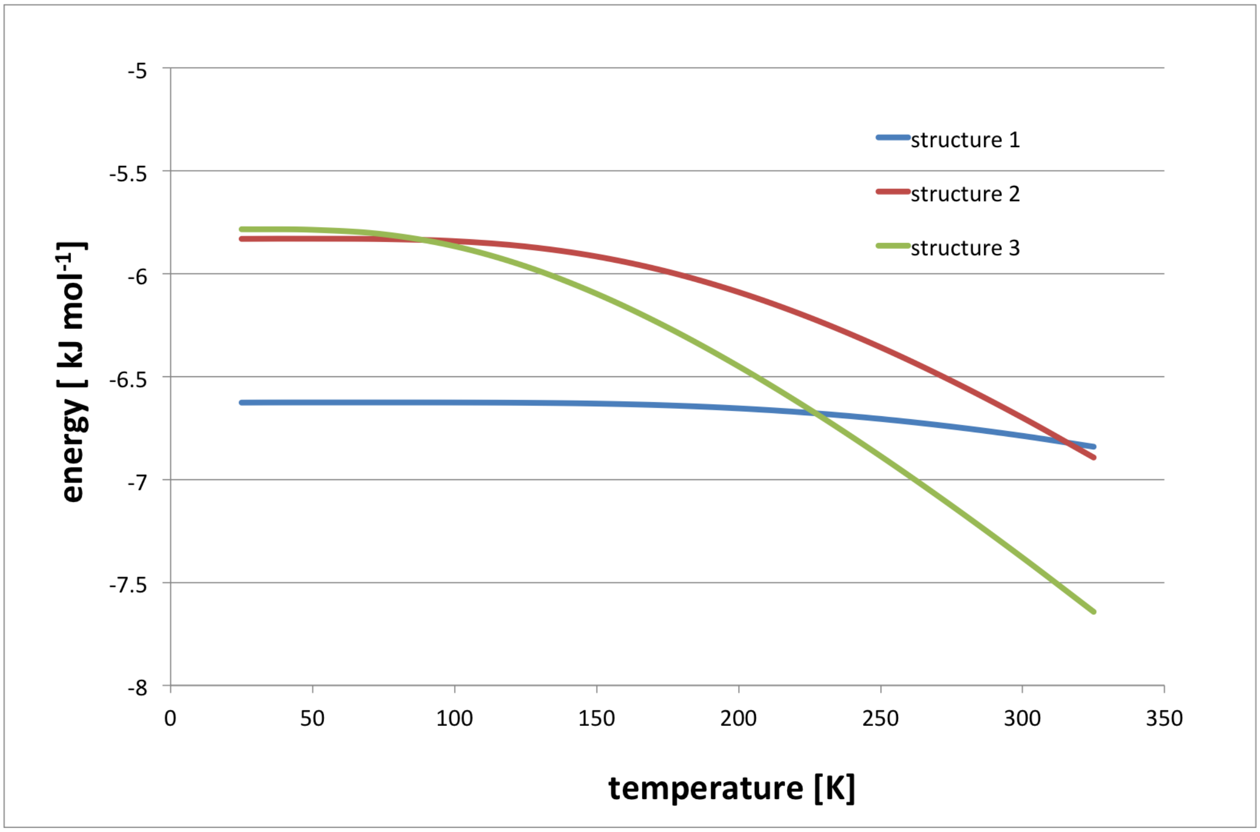

Supplementary Figure 2. Calculated configurational free energy as a function of temperature of the disorder models of predicted structures 1, 2 and 3. Structures 1-3 are the three lowest-energy structures, see Supplementary Tables 1 and 2 for details. Structure 1 corresponds to experimental form B, structure 3 to form C; structure 2 is very similar to structure 3 but has not been observed experimentally in agreement with the computed configurational free energy.

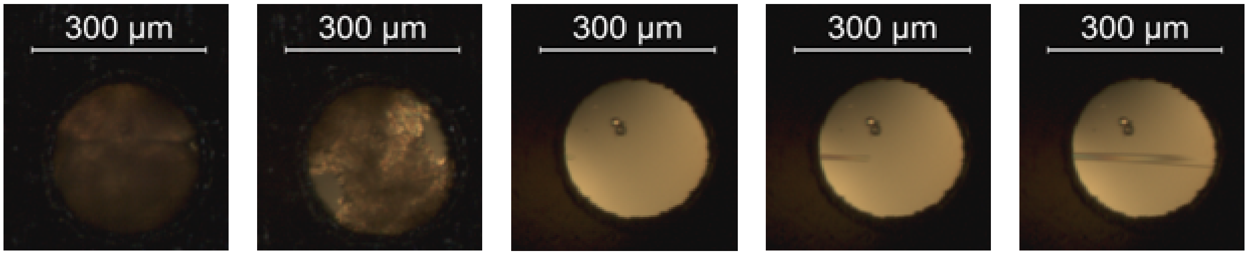

0.45 GPa | 0.15 GPa, heating | < 0.1 GPa, cooling h: 18:27 | Crystal growth h: 18:31 | Crystal growth h: 18:37

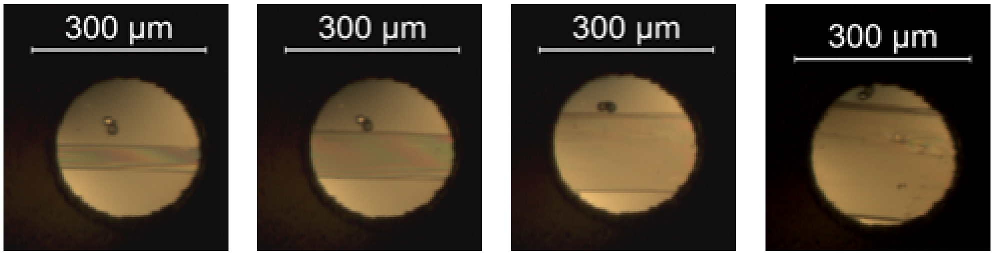

Crystal growth h: 18:50 | Crystal growth h: 19:18 | Crystal growth h: 19:50 | Crystal growth end, one day later, 0.02 GPa

Supplementary Figure 3. Optical images illustrating crystallization and crystal growth stages of Dalcetrapib form C in the DAC. Crystallization and crystal growth stages of Dalcetrapib form C in the DAC with details of pressure and temperature regimes as well as elapsed time.Two ruby chips for *in situ* pressure calibration are also visible in the gasket.

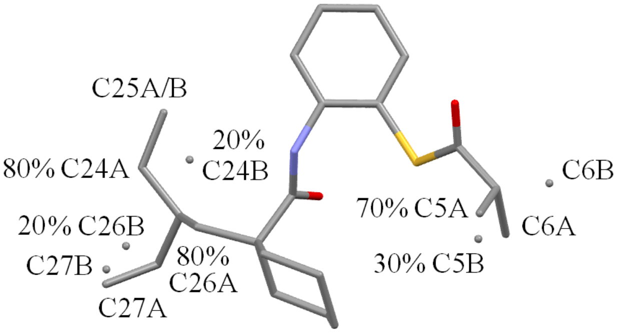

Supplementary Figure 4. Conformation of form C from single-crystal X-ray diffraction at *ca.* 0.02 GPa. Numbering scheme and site occupancy factors for the disordered groups in the experimentally-determined form C. H-atoms have been omitted for clarity.

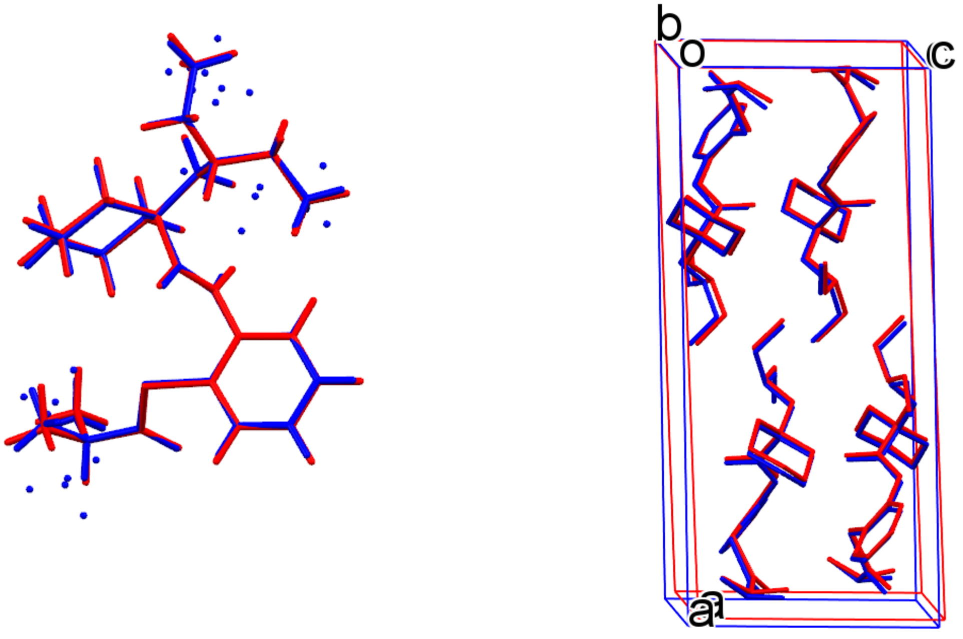

Supplementary Figure 5. Molecular and structural overlays of experimental and computational form C. Left: molecular overlay for all non-disordered and major-disordered component atoms of the experimental (blue) and computational (red) structures (RMSD 0.062); the minor disordered component is depicted for the experimental structure. Right: unit-cell packing of the structures in which H-atoms have been omitted for clarity and only the major disordered component is shown.

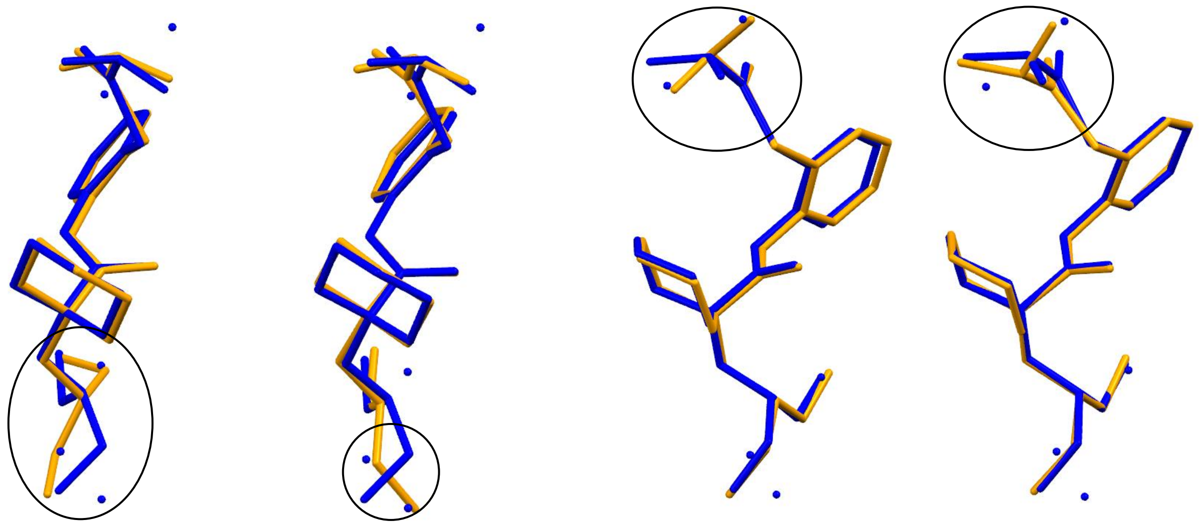

Orange = configuration 2
Blue = form C at high pressure
Population: 22%
Experimental: 0% *

a

Orange = configuration 5
Blue = form C at high pressure
Population: 3%
Experimental: 0% *

b

Orange = configuration 3
Blue = form C at high pressure
Population: 16%
experimental: 30%

c

Orange = configuration 4
Blue = form at high pressure
Population: 6%
experimental: 0

d

Supplementary Figure 6. Molecular overlays of the high-pressure experimental form C and disordered configurations of structure 3 from the computational study. See Supplementary Table 2 for details of the configurations of structure 3. H-atoms have been omitted for clarity. The populations of the minor disordered components found in the computational study and experimentally are also given in percentage.

***** Note that, experimentally, the minor (20%) 3-pentyl group disordered component corresponds to an average of the computed configurations 2 and 5 structures (**a** and **b** in the Figure above, respectively).

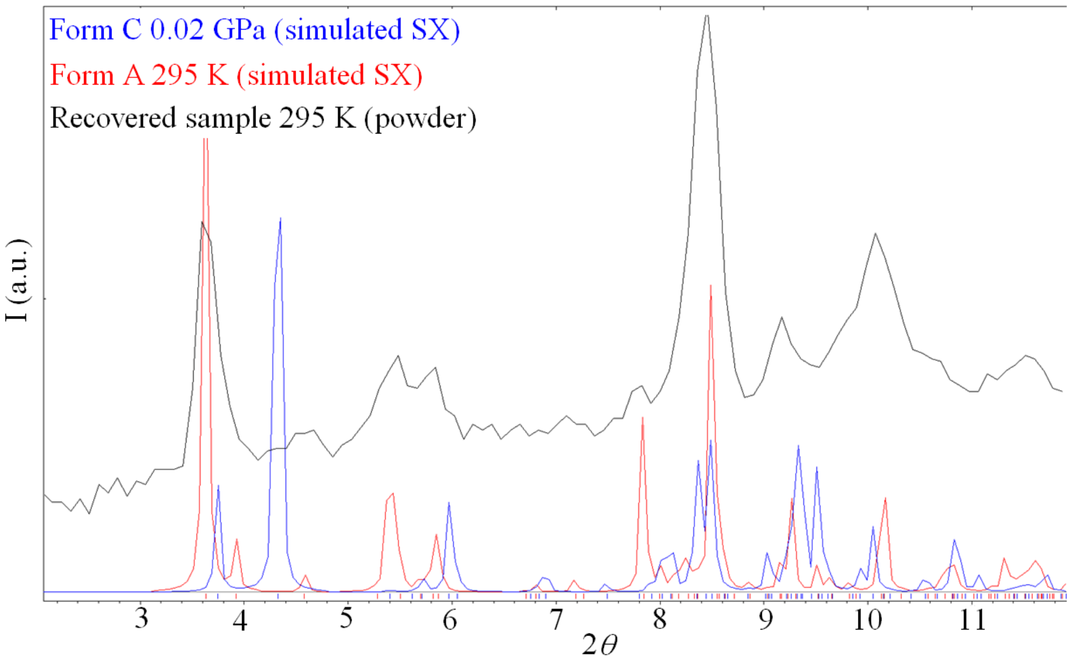

Supplementary Figure 7. Experimental X-ray powder pattern (black) of the recovered sample in Supplementary Fig. 8 taken from a still frame. No background was subtracted. The peak at 2θ *ca.* 10° is possibly contaminated by λ/2 diffraction of the steel gasket (111) reflection (fcc structure). The simulated powder patterns of forms A and C from the single-crystal (SX) structures are also shown for comparison; λ = 0.71073 Å.

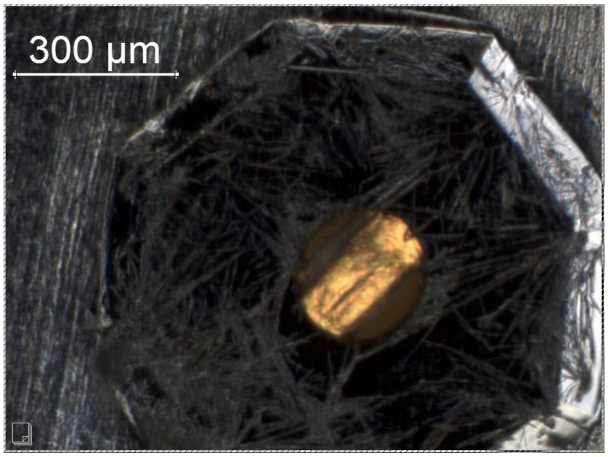

Supplementary Figure 8. Optical image of the recovered crystal in the DAC gasket hole.

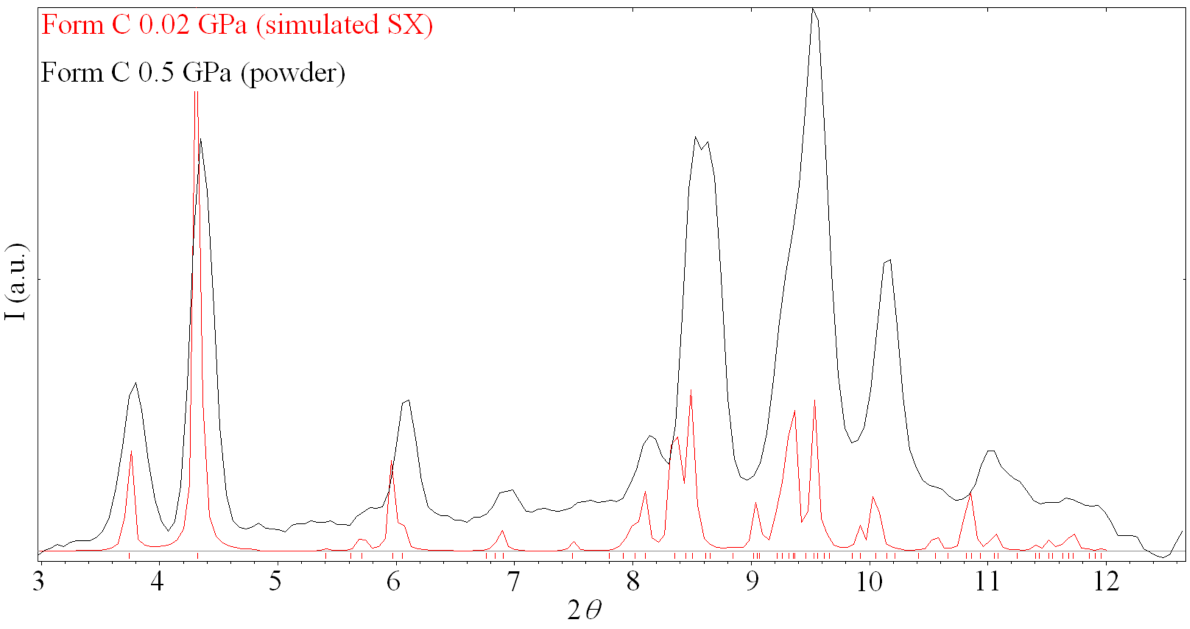

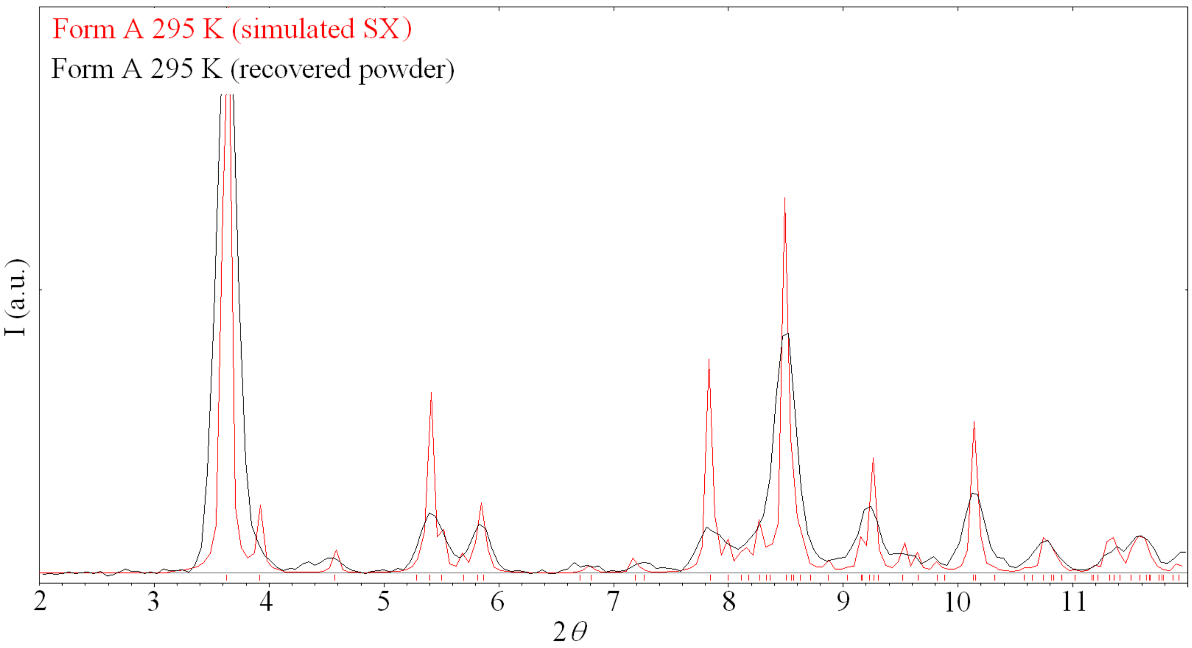

Supplementary Figure 9. Experimental (black) and simulated (red) X-ray powder patterns of form C (top) and form A (bottom) as obtained from powder diffraction and single-crystal data, respectively. λ = 0.71073 Å. Form C was crystallized from solution in the DAC while form A was subsequently obtained in the recovery experiment.

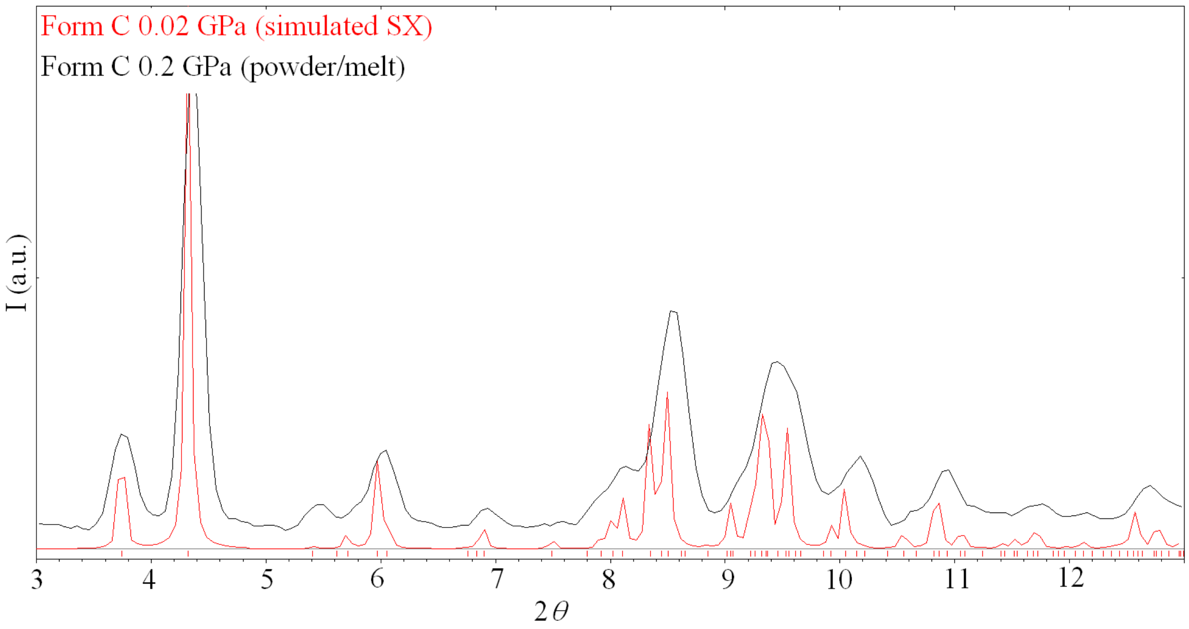

Supplementary Figure 10. Experimental (black) and simulated (red) X-ray powder patterns of form C and form A as obtained from powder diffraction and single-crystal (SX) data, respectively. λ = 0.71073 Å. Form C was crystallized from the melt *in situ* in the DAC.

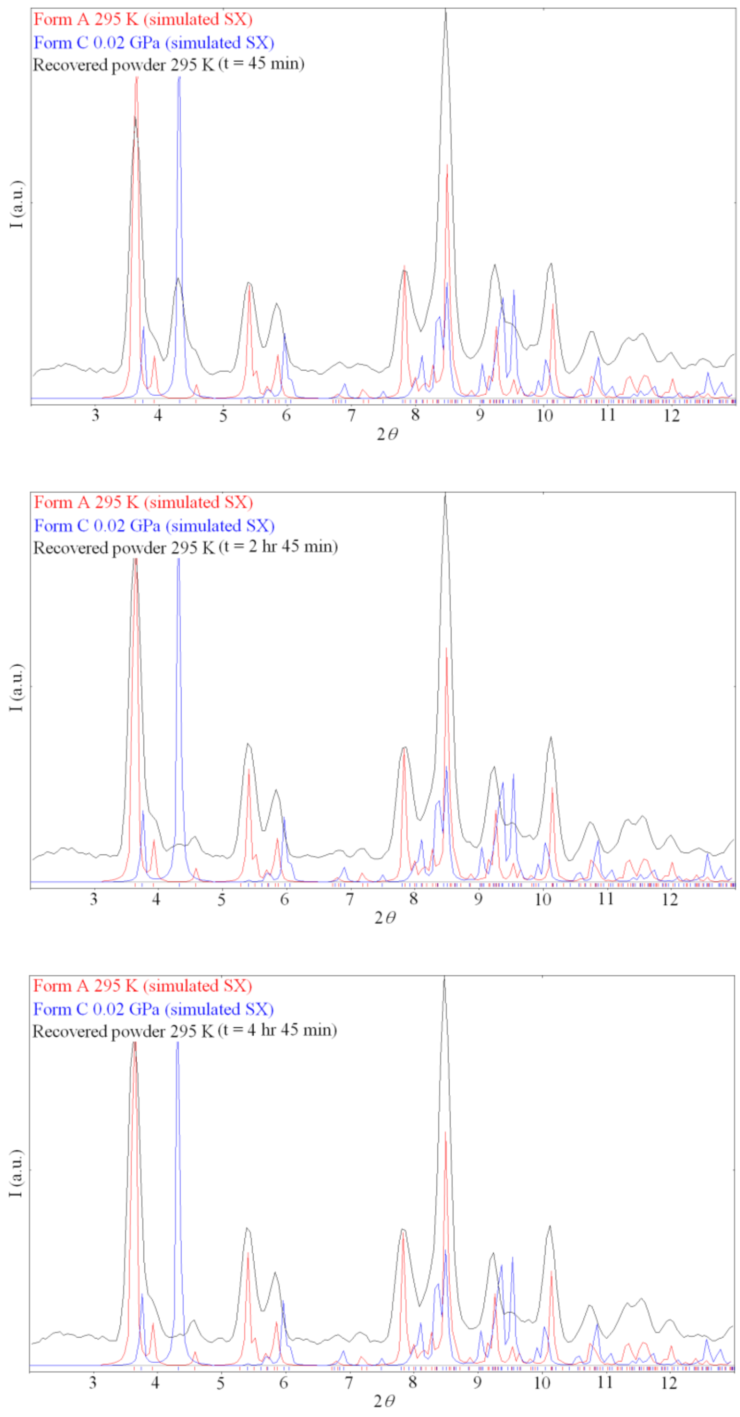

Supplementary Figure 11. Experimental (black) X-ray powder pattern of the recovered sample as a function of time elapsed since depressurization to ambient pressure. Simulated powder patterns of pure forms C (red) and A (blue) as obtained from powder diffraction and single-crystal (SX) data, respectively, are also shown for comparison; λ = 0.71073 Å.

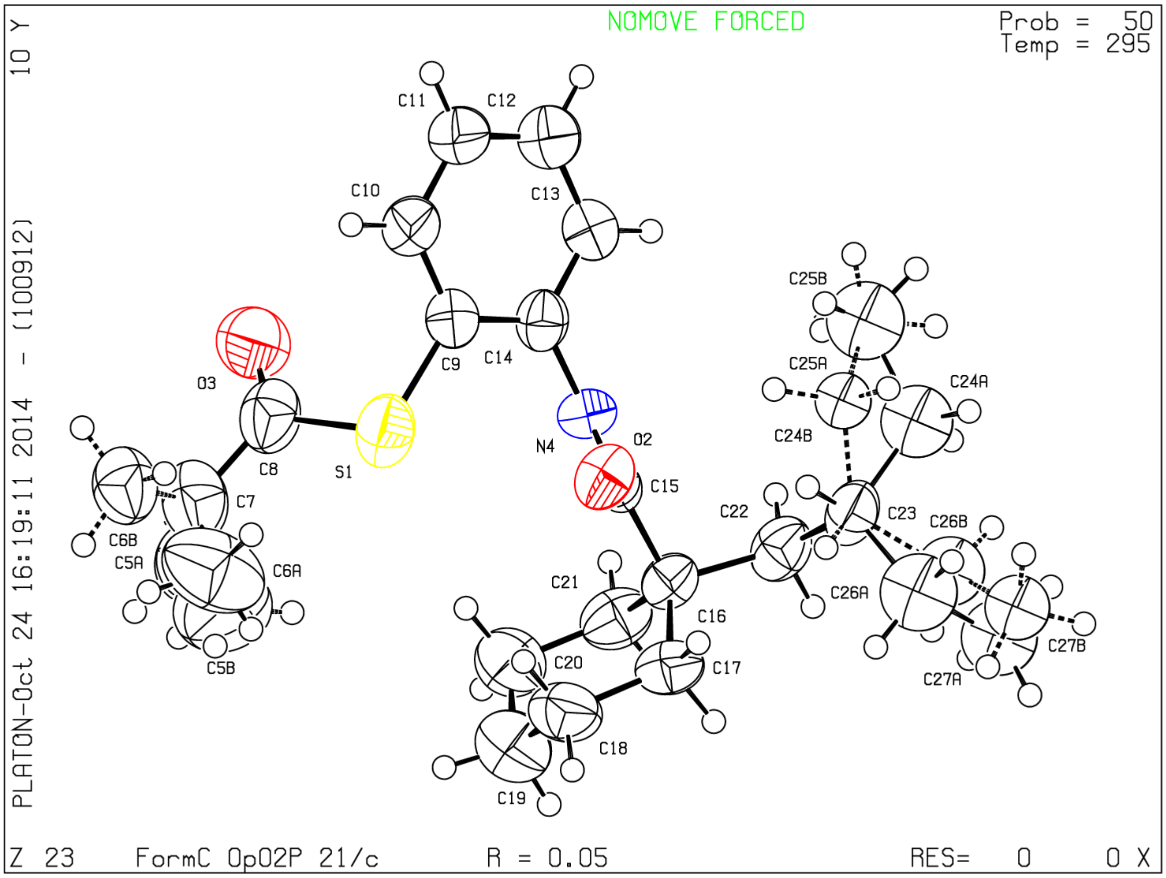

Supplementary Figure 12. ORTEP plot for form C.

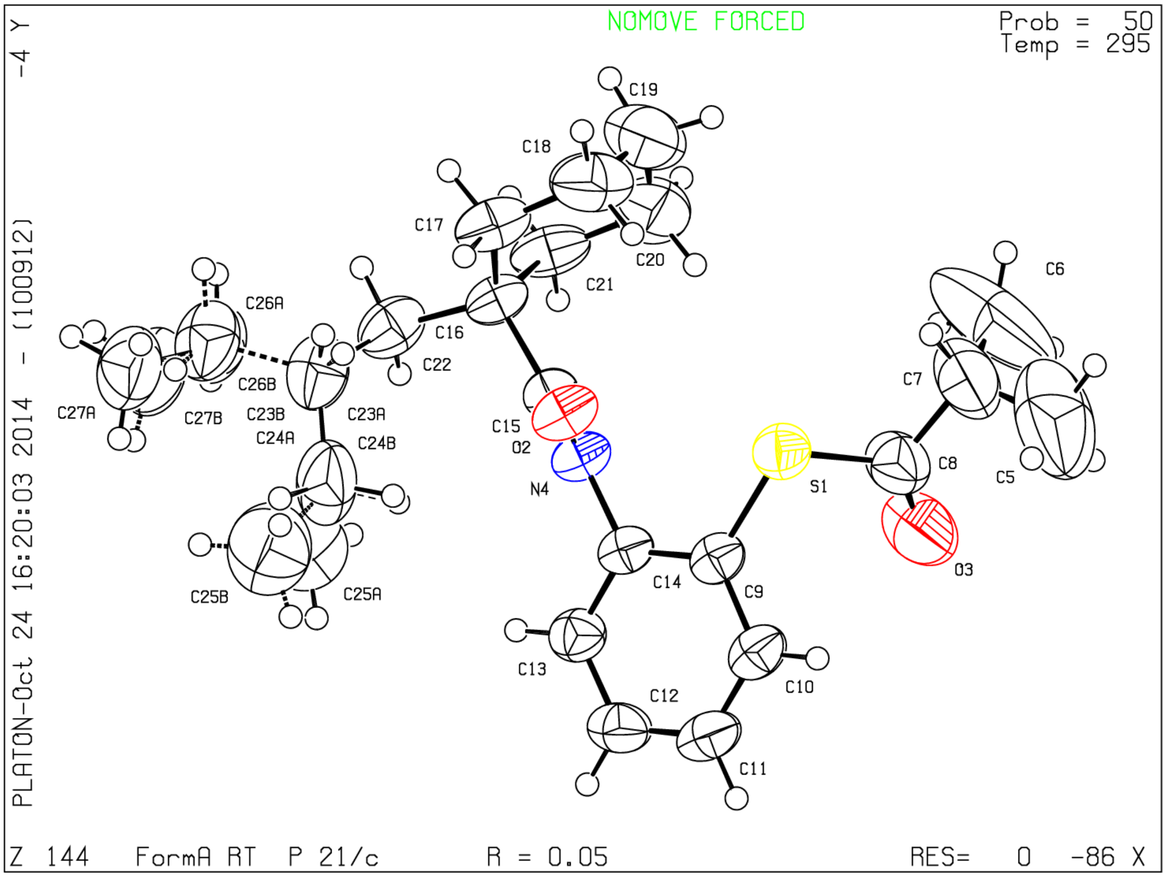

Supplementary Figure 13. ORTEP plot for form A at 295 K.

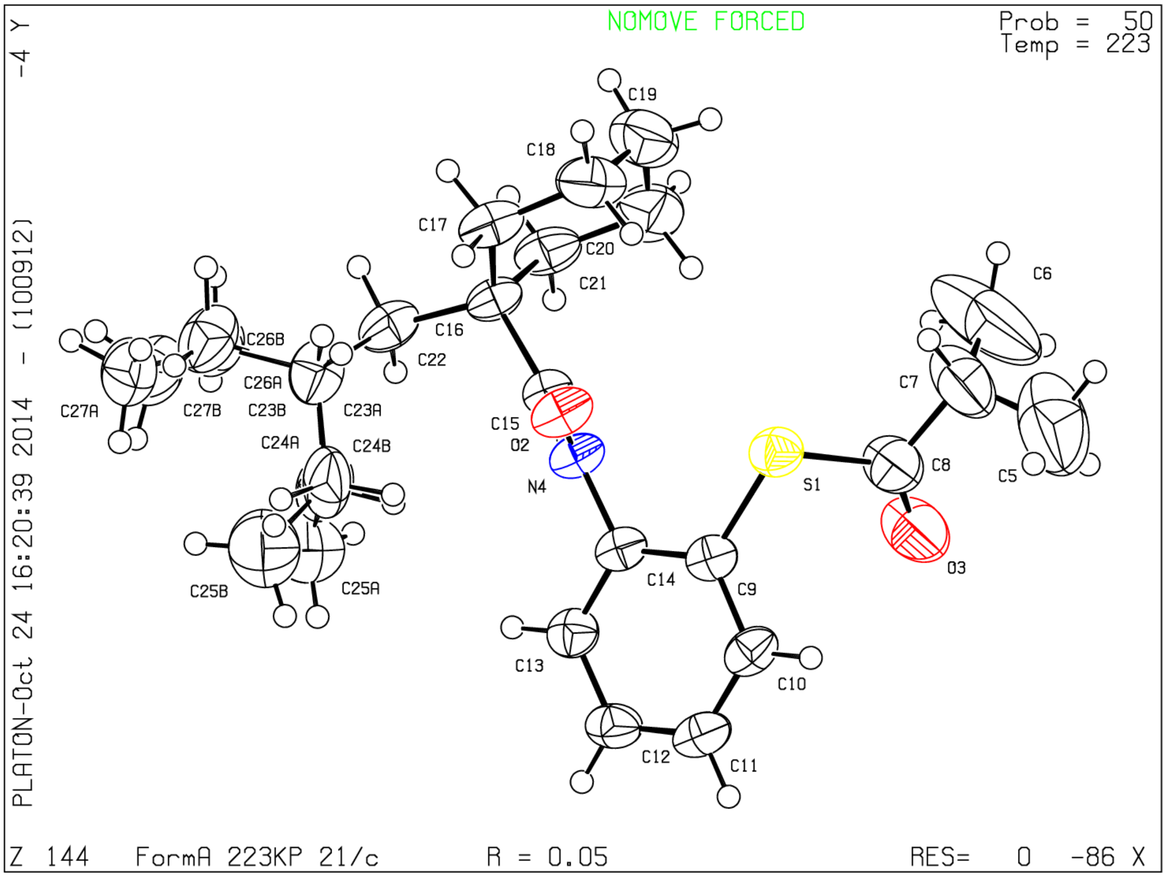

Supplementary Figure 14. ORTEP plot for form A at 223 K.

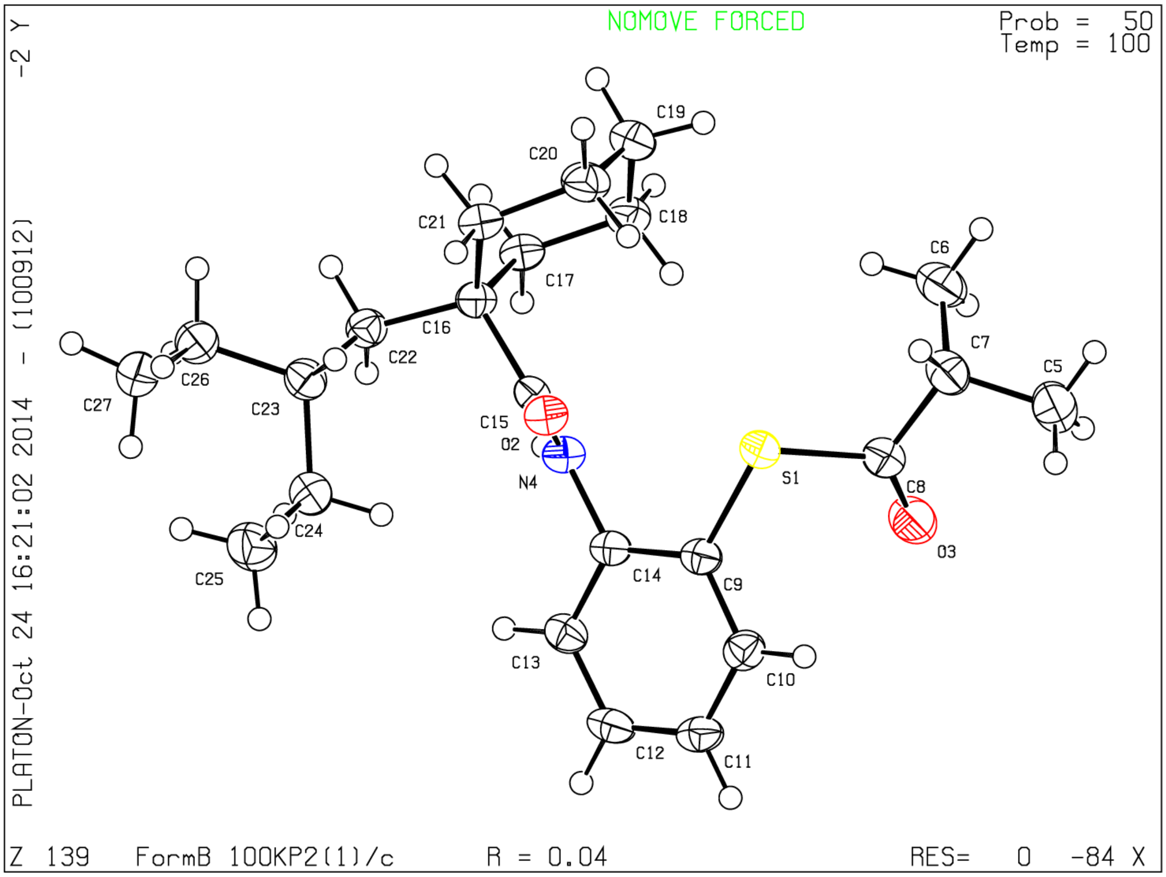

Supplementary Figure 15. ORTEP plot for form B.

Supplementary Table 1. Lattice energies per molecule calculated with BYLP-Grimme and PBE-Neumann-Perrin for the predicted structures of Dalcetrapib. For each method, a constant offset has been applied such that the average energy is zero.

Rank with BPLY –D3	BLYP-D3 [kJ mol^{-1}]	BPE-Neumann-Perrin [kJ mol^{-1}]
1	-6.625020912	-4.65720595
2	-5.82923499	-5.709808532
3	-5.783397597	-4.767768318
4	-4.148789966	-2.548519734
5	-4.05810093	-1.71623657
6	-3.749664818	-0.63321375
7	-3.537858186	-2.20774033
8	-2.618153062	1.911149504
9	-2.442305567	-1.74533193
10	-2.070418259	-0.915516676
11	-1.951609395	-2.050469518
12	-1.49878679	-1.882937396
13	-1.492431294	-0.67607745
14	-1.341767127	-2.832199494
15	-1.021086958	-2.665290844
16	-0.822354489	0.229281828
17	0.388095121	1.0352233
18	0.637541853	1.355817798
19	1.132725785	1.27980617
20	1.170469649	3.988636142
21	1.241677145	0.22263146
22	1.912895345	-2.678253866
23	2.055855093	4.282395366
24	2.543204903	1.886860074
25	2.974553714	0.040603614
26	3.622918069	3.072755774
27	3.87716385	4.182016374
28	4.271334306	2.301053306
29	7.551626288	6.327357548
30	7.596140701	5.571060034

Supplementary Table 2. Disordered configurations of predicted structures 1, 2 and 3 of Dalcetrapib within 8.0 kJ mol^{-1} from the ordered configuration (configuration 1). For all three structures, the energies are with respect to the energy of the ordered state.

**Structure index**	**Configuration index**	**Found by Monte Carlo**	**Found by substitution**	**Substituted from**	**Energy [kJ mol^{-1}]**	**Occupation at 293 K**
1	1	-	-	-	0.00	0.941
1	2	yes	yes	6	6.75	0.059
2	1	-		-	0.0	0.715
2	2	yes	-	-	3.63	0.161
2	3	yes	-	-	5.61	0.071
2	4	yes	-	-	6.34	0.053
3	1	-	-	-	0.0	0.535
3	2	yes	yes	9	2.18	0.219
3	3	yes	yes	13	2.91	0.162
3	4	yes	yes	12	5,72	0.061
3	5	yes	yes	2	6,75	0.033

Supplementary Table 3. Configurations of a hypothetical disorder model of Dalcetrapib form A.

**Name**	**Energy [kJ mol^{-1}]**
form_A_hypothesis_configuration_1	0.0
form_A_hypothesis_configuration_2	-0.42
form_A_hypothesis_configuration_3	0.52
form_A_hypothesis_configuration_4	2.79
form_A_hypothesis_configuration_5	5.72
form_A_hypothesis_configuration_6	5.51

Supplementary Table 4. Structural data for the experimental single-crystal structures of Dalcetrapib.

Structure	**Form C**	**Form A**	**Form A**	**Form B**
CCDC Number	1024144	1024145	1024146	1024147
Supplementary Data	1	2	3	4
Formula	C_{23} H_{35} N O_2 S	C_{23} H_{35} N O_2 S	C_{23} H_{35} N O_2 S	C_{23} H_{35} N O_2 S
Space group	$P2_1/c$	$P2_1/c$	$P2_1/c$	$P2_1/c$
a/Å	21.7729(8)	11.2235(3)	11.181(15)	10.697(2)
b/Å	10.4524(9)	9.8526(2)	9.809(13)	9.840(2)
c/Å	9.7642(3)	20.7922(5)	20.71(3)	20.854(4)
β/°	88.212(3)	88.7400(10)	88.545(13)	90.15(3)
V/Å^3	2221.02(29)	2298.66(9)	2270(5)	2194.9(8)
Z'	1	1	1	1
Density$_{calc}$/g cm^{-3}	1.165	1.126	1.140	1.179
Pressure	0.02 GPa	0.1 MPa	0.1 MPa	0.1 MPa
Temperature/K	295(2)	295(2)	223(2)	100(2)
Measured/unique/ observed reflections	10422/1287/974	17030/3894/3112	7525/2399/1798	14955/3051/2675
R_{int}	0.0378	0.0221	0.0648	0.0522
Parameters/restraints	243/199	283/119	282/278	252/0
θ_{max}/°;completeness/%	20.83/55	24.72/99	21.00/99	57.96
R_1 [$F > 4\sigma$(F)]	0.0533	0.0517 (0.0534)[1]	0.0500	0.0391
wR_2 [all data]	0.1554	0.1564 (0.1674)[1]	0.1397	0.1037

[1] values outside and inside the brackets refer to occupancies of the disordered 3-pentyl side chain fixed to 60:40 and 50:50, respectively; see text for details.

Supplementary Note 1

Energy calculations

All DFT-D lattice energy optimizations have been carried out with the GRACE program. For the calculation of DFT energies and forces, GRACE calls the ab-initio total-energy and molecular-dynamics program VASP developed at the "Institut für Materialphysik" of Vienna University[1-4]. The dispersion correction is implemented in GRACE.

The energy calculation method BLYP-D3 combines the BLYP functional, implemented in VASP, with the dispersion correction according to Grimme[5], implemented in GRACE. The energy method PBE-Neumann-Perrin combines the PBE functional with the dispersion correction according to Neumann-Perrin[6].

DFT calculations use a plane wave cutoff energy of 520 eV and a *k*-point spacing if roughly 0.07 $Å^{-1}$. All lattice energy minimizations have been converged to within at least 0.003 Å for atomic displacements, 0.001 kJ mol^{-1} per atom for energy changes, 1.7 kJ mol^{-1}/Å for atomic forces and 0.0125 kbar for cell stress.

Lattice energies calculated with BLYP-D3 and PBE-Neumann-Perrin

The lattice energies of the 30 most stable predicted crystal structures according to BLYP-D3 are listed in Supplementary Table 1 both for BLYP-D3 and PBE-Neumann-Perrin. On average, the lattice energies calculated with both methods deviate from each other by 1.9 kJ mol^{-1} per molecule. The 30 structures resulting from the BLYP-D3 optimization are supplied as Supplementary Data 5 – 34 (see Supplementary Note 3).

Disorder models

Considering the possibility of disorder significantly complicates the tasks of crystal structure prediction. Therefore, the blind test compounds have always been selected as to avoid disordered structures and typically compounds with ordered crystal structures are chosen for CSP validation studies. However, disorder is a commonly observed phenomenon in molecular crystals and can have a strong impact on the relative stability of crystal forms because of its contribution to the crystal free energy. With two disordered crystal polymorphs out of three, Dalcetrapib exhibits a particularly high degree of disorder and the energy ranking presented here would have been incomplete without an attempt to quantify the configurational contribution to the crystal free energy by means of lattice energy calculations.

Our aim is to predict disorder that is an equilibrium property of a crystal form and contributes to the crystal free energy. For this type of disorder we will use the term thermodynamic disorder. Crystallographers make a distinction between dynamic versus static disorder, according to whether atoms move on the timescale of a diffraction experiment or not. For samples in thermodynamic equilibrium, dynamic disorder actually is thermodynamic disorder. Static disorder typically is not thermodynamic disorder and may originate from stacking faults or unfavorable molecular conformations build into the crystal during the growth process. However, energy landscapes with high energy-barriers but energetically close energy minima are conceivable for which static disorder would actually be thermodynamic disorder.

Thermodynamic disorder occurs when local atomic rearrangements are possible at an energetic cost of not much more than kT, where k is the Boltzmann constant and T is the temperature. Because a local rearrangement applied to all molecules in the unit cell of an ordered structural model leads to another ordered structural model, the presence of families of similar structures among the first few predicted structures with energy differences less than $3kT \approx 7.5$ kJ mol^{-1}

(room temperature) per molecule must be considered as a strong indication for a possibly disordered crystal structure. However, the presence of a family is not a necessary condition, since a local atomic rearrangement may be energetically feasible for a single molecule surrounded by molecules in the original configuration, but not for all molecules at once. It is also not a sufficient condition, because the energetic cost for the change of a molecular conformation may be low for a concerted rearrangement of a one-, two- or three-dimensional net of molecules, but not for a single molecule. A pragmatic approach is to search for thermodynamic disorder whenever a family of similar and energetically close structures is found.

To find the configurations that contribute to a disordered crystal structure, two different strategies have been tried for the predicted structures 1, 2 and 3. The first approach, called the substitution method, is to change the conformation of one molecule in a 1×1×1 super cell to another conformation found in the same family of similar structures. Full lattice energy minimizations at the BLYP-D3 level of theory are carried out for all substituted structures. In the second approach, called the Monte Carlo method, the crystal structure generation engine is used in a special disorder location mode. At all stages of the crystal structure generation, 66% of the atoms in a 1×1×1 super cell had to stay within 1.0 Å from their starting positions. For a crystal structure to be accepted as a final result, 75% of all atoms at to be within 0.5 Å from their starting positions and the region of change had to form a zero-dimensional island. In the structure generation, lattice energies were calculated with the tailor-made force field from the crystal prediction study and structures were searched in an energy window of 22 kJ mol^{-1}. All structures within 15 kJ mol^{-1} from the bottom of the window were lattice energy optimized at the BLYP-D3 level of theory. Structures within 7.5 kJ mol^{-1} from the ordered state are listed in Supplementary Table 2. For structure 2, only the Monte Carlo method has been applied. For structures 1 and 3, the Monte

Carlo method and the substitution method have been used and yield the same results, thus validating the validity of both approaches.

Assuming that all molecules in a crystal are disordered independently, the thermal occupancies of the configurations and their enthalpy and free energy contributions are readily evaluated using textbook formulas[7].

The partition function is defined as:

$$Z_i = \sum_j \exp(-\Delta E_{i,j} / kT) \tag{1}$$

Here *i* refers to the structure index and *j* refers to the configuration index. $\Delta E_{i,j}$ is the energy difference with respect to configuration 1 that can be taken from Supplementary Table 2.

Occupancies can be obtained as follows:

$$p_{i,j} = \frac{\exp(-\Delta E_{i,j} / kT)}{Z_i} \tag{2}$$

The free energy is related to the partition function:

$$F_i = E_i - kt \ln Z_i \tag{3}$$

Here the lattice energy of the ordered state, E_i, has been added explicitly. It can be found in Supplementary Table 1.

At ambient pressure, the enthalpy is practically identical to the average energy:

$$H_i = E_i + \frac{\sum_j \Delta E_{i,j} \exp(-\Delta E_{i,j} / kT)}{Z_i} + pV_i \tag{4}$$

Using the above formulas and the energy values from Supplementary Tables 1 and 2, the occupancies in Supplementary Table 2 and the free energies curves for the disorder models of structures 1, 2 and 3 in Supplementary Fig. 2 have been obtained.

In terms of free energy, the disordered structure 3 is more stable than the disordered structure 2 at room temperature, which is in agreement with the fact that the disorder model of structure 3 matches the experimentally observed form C very well. Structure 1 is only slightly disordered. The most favorable atomic rearrangement (configuration 2) has a thermal occupation of less than 1.3% below the temperature of the form A – form B phase transition at -87 °C. Below the phase-transition temperature, structure 1 is more stable than structures 2 and 3 even if the configurational free energy contribution is taken into account.

The nature of the disorder in form A has turned out to be difficult to understand. The experimental phase transition enthalpy is about 3.3 kJ mol^{-1}. At the phase-transition temperature, the free energy difference is zero, such that the entropy difference can be derived from the phase transition enthalpy:

$$\Delta S_{A,B} = \frac{\Delta H_{A,B}}{T} = k * \ln \frac{N_A}{N_B} \qquad (5)$$

Relating the entropy to a number of states, it follows that per molecule form A has 8 times more states than form B. It turned out that none of the predicted structures of the structure-1 family could accommodate a substantial amount of disorder. Since the crystal structure prediction study had been carried out for one molecule per asymmetric unit only, an attempt was made to find structures with two molecules per asymmetric unit also belonging to the structure-1 family that may be able to accommodate the required amount of disorder. In a first step, the Monte Carlo disorder search tool was applied to structure 1, not limiting the regions of structural change to

zero-dimensional islands. The space-group symmetry of the obtained disordered models was analyzed, and for structures with two molecules per asymmetric unit the Monte Carlo disorder search tool was applied again, this time limiting the regions of change to zero-dimensional islands. Only one structural model with a substantial amount of disorder could be identified this way. Its configurations are listed in Supplementary Table 3. The lattice energies of all configurations have been minimized at the BLYP-D3 level of theory.

Configuration 1 has *P*-1 symmetry and a lattice energy of -3.1 kJ mol^{-1} per molecule, *i.e*. 3.6 kJ mol^{-1} more than structure 1. Since it has two molecules per asymmetric unit, disorder can potentially occur at the propyl or 3-pentyl groups of both molecules, *i.e*. at four different sites. For one of the molecules, the 3-pentyl side chain can adopt four different states (configurations 1, 2, 3 and 4). One of these states involves a reorientation of a neighboring propyl group. For the other molecule, the 3-pentyl side chain is not disordered. In addition, a concerted reorientation is possible for two pairs of propyl groups (configurations 5 and 6). With the listed configurations, we are far from the expected count of 8 states per molecule on average. However, this does not necessarily invalidate the structural model, since there may also be a significant vibrational contribution to the free energy. A structural model that can accommodate a significant amount of configurational disorder is also likely to allow for a certain number of low frequency vibrations. The lattice energy difference between structure 1 and configuration 1 of the hypothetical disorder model is close to the experimentally observed value of 3.3 kJ mol^{-1}. The evaluation of the vibrational contribution to the free energy at the BYLP-Grimme level of theory has not been possible within the amount of CPU time available for the study.

CPU time consumption

The crystal structure prediction study, including the generation of a tailor-made force field, required 16.5 weeks of CPU time on a LUNIX cluster featuring 64 quad core AMS Opteron processors with 2 GB RAM per core and connected by an InfiniPath network. The disorder analysis required 6 additional weeks of CPU time on the same cluster.

Structural models

All predicted structures and disorder models mentioned in the main text or the supplementary information are supplied as Supplementary Data (see Supplementary Note 3).

Supplementary Note 2

Experimental procedures

Form C

High-pressure crystallization and crystal growth

The compound was used as received. A modified Merrill-Bassett diamond-anvil cell (DAC)[8] equipped with 800 μm culet diamonds mounted on WC seats and Inconel steel gaskets of 300 μm diameter was used for all experiments. The opening angle of the cell for X-rays was 84°. The ruby fluorescence method[9] was used to monitor the pressure inside the DAC using an in-house built instrument with an accuracy of 0.05 GPa.

At conditions of ambient pressure, crystallization of Dalcetrapib is known to be kinetically hindered and solutions can be easily supersaturated. By using a 0.82 M THF solution, crystallization of polycrystalline material was observed after *ca.* 24 hours from initial loading, at

a pressure of *ca.* 0.45 GPa. The time lag is a sign that under these conditions crystallization is also kinetically driven. Single crystals are usually grown by temperature and/or pressure cycling of the polycrystalline material until one seed is left, at which point the cell is allowed to cool slowly by removing the heat source and leaving the DAC under the microscope on a glass support. Gentle heating (323-333 K) is preferred for organic compounds to avoid compound decomposition. In this experiment, the polycrystalline material could not be dissolved at 0.45 GPa. The optimal pressure for crystal growth and gentle heating was below 0.1 GPa; the pressure inside the sample chamber could not be determined reliably during the initial stages of crystal growth. During crystal growth the pressure inside the chamber drops significantly, indicating that the solid is considerably denser than the solution. The final pressure inside the chamber was *ca.* 0.02 GPa. Despite the large, intrinsic, error associated with the pressure measurement, which is exacerbated by the fact that the ruby fluorescence pressure scale has been calibrated for much higher pressures, our experience with the in-house built instrument and calibration procedure enables us to state the pressure was well below 0.1 GPa and yet above ambient pressure, as later confirmed by the recovery experiments and as demonstrated by the absence of air bubbles, present on loading. Stages of crystallization and crystal growth are depicted in Supplementary Fig. 3.

High-pressure crystallography

In situ single-crystal X-ray diffraction of form C at 0.02 GPa

Single-crystal X-ray diffraction data were collected at ambient temperature on a Bruker Apex II diffractometer equipped with Mo sealed tube radiation. A standard data collection strategy that aims at optimizing data redundancy and completeness was used. To further increase completeness, two data sets with the DAC in two different orientations were collected. The

scattering fall off at a resolution greater than 1.0 Å gave a first good indication that the structure is disordered. For data reduction, a standard strategy was used[10].

The structure was solved by direct methods using the program SIR92[11]. Full-matrix least-squares structure refinement against F^2 was performed using the program SHELXL[12] (Version 2013/3) through the GUI interface SHELXLE[13].

Refinement was carried out using two unit-cell settings:

- a = 21.7729(8) b = 10.4524(9) c = 9.7642(3) β = 88.212(3), space group $P2_1/c$, which corresponds to the unit cell of structure 3 from the crystal structure prediction study.
- a = 21.7729(8) b = 10.4524(9) c = 9.7642(3) β = 91.788(3), which is the conventional monoclinic setting for space group $P2_1/c$.

Both propyl and 3-pentyl side chains are disordered over two positions (Supplementary Fig. 4); site occupancies were initially refined and later fixed to the refined values. Modeling of disorder has a significant effect on the *R*-factor, which increases to a value of over 7% when disorder is not taken into account. Restraints on 1,2 and 1,3 bond distances were used to ensure a chemically meaningful geometry; Anisotropic displacement parameters (ADPs) were also refined subject to enhanced rigid-body restraints (RIGU)[14]; the disordered ethyl groups were refined isotropically and for some atoms positions and U_{iso} constraints were used to enforce a chemically-reasonable model. All H-atoms were refined with a riding model. A final *R*-factor of 5.3 % is extremely satisfactory for this structure and the given data completeness and resolution. Further crystallographic details are given in Supplementary Table 4 and Supplementary Data 1.

Comparison of experimental and computational structures

From comparison of the unit-cell constants, molecular geometry and crystal packing, the experimentally found form C high-pressure structure shows an excellent correspondence with structure 3 from the computational study. An overlay of the two structures is shown in Supplementary Fig. 5.

The experimentally determined disorder matches the results of the disorder calculations quite well, both in terms of geometry and populations of the propyl and 3-pentyl side chains. The computed major disordered components populations are 79% (configurations 1, 2 and 5) and 76% (configurations 1, 3 and 4) for the propyl and 3-pentyl groups, respectively (*cf.* experimental values of 70% and 80%). The calculations indicate the presence of further conformers with less than 8% occupancy and with atomic positions very close to the two main components of disorder. Although in theory the results of the calculations could be used to build more accurate refinement models, in practice, the limited amount of data that can be collected with the DAC precludes this without running into overparameterization and severe parameter correlation problems. It should be noted that in the case of the 3-pentyl group, the experimental model is actually an average of the computed configurations 2 and 5 (Supplementary Fig. 6a and 6b).

Recovery of form C to conditions of ambient temperature and pressure

Recovery of a single-crystal sample

In order to investigate whether the single-crystal sample of form C crystallized at high pressure is also stable at ambient pressure, the pressure inside the DAC was slowly released from 0.02 GPa to ambient pressure and the crystal inspected visually under an optical microscope. The crystal was observed to dissolve but remained edged in between the gasket once the cell was opened. The gasket was quickly mounted on the single-crystal diffractometer at ambient pressure. No

single-crystal diffraction was observed from the sample but a diffraction shot was recorded at fixed sample position (Supplementary Fig. 7). The quality of the diffraction pattern is very poor, no background was subtracted and the pattern can be at best used for semi-qualitative phase identification purposes. A comparison of 2θ values and intensities with those of the simulated A and C forms appears to favor the presence of form A. The barrier to molecular rearrangement associated with a solid-state phase transition would be quite substantial: H-bonded chains in form A involve molecules related by a screw axis, whilst in form C molecules are related by glide symmetry; however since form C was surrounded by solution in the DAC, such a barrier to rearrangement would be easily overcome.

On subsequent closer inspection of the recovered crystal under polarized light, more details were revealed (Supplementary Fig. 8). Two features were observed. First, the crystal shows some "finer" non-single-crystal features, which could be indicative of a liquid-assisted transformation to form A; second, and most importantly, fine needle-like crystals are observed on the gasket and on the edge of the gasket hole. These crystals, which were not measured during the diffraction experiment, likely formed on either of the following two instances (or a combination thereof): a) from evaporation of the solution used on cell loading, *e.g.* liquid trapped between the diamond culets and crystallization on the diamond pavilion, presumably yielding form A and b) once the DAC was opened and the liquid escaped from the gasket hole.

Recovery of a polycrystalline sample

In order to better investigate the stability of form C at ambient pressure, the recovery of polycrystalline material crystallized *in situ* at high pressure was investigated. A 0.82 M THF solution was used for this experiment and crystals were obtained at 0.5 GPa. *In situ* diffraction experiments were performed allowing for some sample rotation during exposure to X-rays to

improve the counting statistics and powder averaging. DAC/gasket background subtraction was also performed. The resulting powder pattern, shown in Supplementary Fig. 9, can be clearly attributed to form C. The cell was subsequently cooled for two hours at 298 K and for 30 minutes to 253 K. Once cooled, the cell was rapidly opened, the very modest amount of excess solution on the gasket dabbed with a cotton fiber and the gasket containing polycrystalline material mounted on the diffractometer. No appreciable dissolution was observed on decompression. From the comparison of the powder patterns, it is clear that the decompressed sample corresponds to form A, suggesting that a solution-mediated transition took place on decompression and confirming the single-crystal recovery results.

Crystallization of form C at high pressure from the melt

Recovery experiments of form C crystallized at high pressure from solution indicate that the sample always transforms to form A on decompression *via* a solution-mediated transition. It is possible that seeds of form A outside the sample chamber but already present before decompression (*e.g.* on the edge of the gasket, or on the diamond pavilion) act as seeds. To maximize the chances of successful form C recovery, decompression should take place in absence of a solution. The modest melting temperature of form A at ambient conditions (T_m onset = 64.9 °C from DSC measurements) does in principle allow for an *in situ* high-pressure crystallization experiment from the melt. The melt was loaded in a preheated DAC, excess material was dabbed away and the sample rapidly pressurized to 0.6 GPa. No crystallization was observed to occur on cooling to ambient temperature. The sample remained in the melt state for over a week and subsequently over 1 month after depressurization to 0.3 GPa. The formation of a supercooled melt for this compound has also been observed at ambient-pressure conditions. Cooling of the cell to 298-253 K did not induce crystallization. In order to trigger nucleation the

cell was subjected to heating/cooling cycles in the 293-323 K range. Cycling proved to be crucial for maximizing the amount of crystalline material in the DAC. Crystal growth is sluggish and coexistence of the melt and of the solid was observed after the first cycle. At the end of second cycle, the sample chamber appeared to be fully containing crystalline material. *In situ* diffraction experiments at ambient temperature and 0.2 GPa clearly indicate the presence of form C in the DAC (Supplementary Fig. 10).

The pressure inside the DAC was subsequently decreased to ambient but the cell was not opened to avoid possible contamination with crystallites of form A crystallized outside the sample chamber. The cell was transferred to the diffractometer; diffraction data clearly indicate the presence of both forms A and C (Supplementary Fig. 11, top). Data were collected approximately 45 minutes after the pressure was released to ambient. Two further powder patterns were collected at two hour intervals (Supplementary Fig. 11, middle and bottom). After two hours the sample had almost completely transformed to form A; after a further two hours the sample appeared to have completely transformed.

The results of these experiments indicate that whilst form C is recoverable to ambient pressure and temperature, this form is metastable and form A is the most thermodynamically stable form under these conditions.

Form A

Form A at 295 K

A single-crystal specimen was selected from a batch of form A that was grown from a 0.82 M THF solution at 253 K. Diffraction data were collected at ambient temperature on a Bruker Apex II diffractometer equipped with Mo sealed tube radiation. A standard data collection strategy that aims at optimizing data redundancy and completeness was used.

Both propyl and 3-pentyl side chains are disordered; significant displacement is also observed for the carbonyl oxygen, indicating motion for the whole corresponding side chain. Several structural models were tested for refinement and the one that best describes disorder without overparameterization is reported here. Disordered sites were fixed to half occupancy; when freely refined, occupancies for the 3-pentyl side chain are 0.594(6):0.406(6).

Disorder was not modeled for the propyl side chain: the preference for using an anisotropic model over an isotropic model with fewer parameters to describe large thermal motion has been discussed by Watkin[15]. As in the case of form C at high pressure, modeling of disorder has a significant effect on the *R*-factor. Restraints on 1,2 and 1,3 bond distances were used to ensure a chemically meaningful geometry; ADPs were refined for all non-H atoms, some atoms were further subject to enhanced rigid-body restraints (RIGU)[14]; U_{iso} and position constraints for atoms occupying the same site were used to enforce a chemically-reasonable model. All H-atoms were refined with a riding model. A final *R*-factor of 5.3 % (5.17 % with disorder fixed to 60:40) is satisfactory for this structure. Further crystallographic details are given in Supplementary Table 4 and Supplementary Data 2.

Form A at 223 K

In order to investigate the nature of disorder, *i.e.* static or dynamic, a multi-temperature experiment would ideally be carried out[16]. In the particular case of Form A, since the phase transition to the low-temperature form B takes place at relatively high low temperatures, the temperature window available for such an experiment is modest. Cooling of form A proved to be a challenging task: reflection broadening and crystal splitting occurred, irrespective of cooling rate, with the number of crystalline domains increasing as a function of increasing time, ultimately leading to a complete crystal destruction. Splitting was observed for crystals cooled in

the 223-203 K temperature range. One of the challenges associated with data collection and processing is that as the crystal breaks up, it is no longer centered on the diffractometer, making data integration a non-straightforward task. After several attempts, a partial data collection lasting 7 hours was successfully performed on a specimen at 223 K.

A similar refinement model used for form A at 295 K was employed with the difference that, given the lower data resolution, the use of RIGU restraints was extended to all atoms. Compared to the 295 K data collection, no structural changes are observed at 223 K, as expected. Both propyl and 3-pentyl side chains are still disordered; however, the U_{eq} is significantly reduced for all atoms (reduced thermal motion), in particular for the disordered 3-pentyl side chain. This effect is not ascribed to the use of RIGU restraints, as confirmed in a comparison of a similarly restrained model. Disorder was modeled with equal split occupancies. A careful refinement of the site occupancy factors [0.505(7):0.495(7)] indicates that the refined values for the 295 and 223 K structures are only marginally statistically significantly different, even when the data for the two structures are cut to the same resolution. The refinement appears to suggest there is a dynamic component to the structural disorder, though two data points are insufficient to draw any definitive conclusions. Static and dynamic disorder might also coexist in the structure. A final R-factor of 5.0 % is satisfactory for this structure. Further crystallographic details are given in Supplementary Table 4 and Supplementary Data 3.

Possible reasons for the observed crystal cracking on cooling may be ascribed to the microstructure of form A, *e.g.* the presence of defects, the crystal's stress/strain gradients and its response to thermal stress. These effects are well known in crystals of inorganic compounds and proteins. The effects of temperature annealing were not investigated.

Disorder in form A

The atomic coordinates of the disordered high temperature form A are compatible with a mixture of the predicted structure 1 and structure 22. As far as the crystallographic data can tell, crystals grown at ambient temperature and at 253 K, and measured at room temperature, all show the same type and amount of disorder. This would indicate that the conformational disorder in form A is to a first approximation independent of the crystal growth conditions. The transition from form A to form B is a sharp event, as confirmed by DSC and diffraction experiments. Variable-temperature XRPD experiments (Supplementary Fig. 1) indicate that upon transformation a significant shortening of the *a*-axis (*ca.* 0.5 Å) takes place, whilst above the transition temperature shortening of this lattice parameter is within the expected range of structural contraction on cooling.

From the available X-ray data, it was not possible to distinguish between sheets or microdomains or other types of disorder other than the one modeled. However, based on the available data, the possibility that form A corresponds to a mixture of two distinct polymorphs, echoing the case of aspirin, which was described as "intergrowths of two "polymorphic" domains"[17], seems unlikely, although in contrast to the case of aspirin, the lattice parameters of Dalcetrapib forms A and B are rather similar, which would lead to overlapping reflections. No signal for diffuse scattering indicative for stacking disorder was detected. Additionally, whilst in the case of aspirin crystals with a varying degree of intergrowth could be obtained, the analyzed crystals of Dalcetrapib form A appear to lead to the same type of diffraction and to the same structural features. A more extensive screening of form A crystals would be necessary to confirm this. The observed crystal cracking on cooling results in the buildup of several domains. Indexing of the domains from the single-crystal data did not point out to significant differences in unit-cell parameters, indicating that all domains belong to the same one form. This is further proof that upon cooling there is no

gradual transition from a mixed form A/B form to a pure form B but rather that the transition is a sharp one.

Form B at 100 K

A single-crystal specimen was selected from a batch of form A. Diffraction data were collected at 100 K on an Agilent Technologies Xcalibur diffractometer equipped with an Enhance Ultra (Cu) X-ray source and a Ruby CCD detector. Data were collected on a fragment of form A mounted on the diffractometer that survived the low-temperature phase transition.

The structure is fully ordered. ADPs were refined for all non H-atoms. All H-atoms were refined with a riding model with the exception of the amide H-atom, for which position and U_{iso} were freely refined. Further crystallographic details are given in Supplementary Table 4 and Supplementary Data 4.

Responses to CheckCIF alerts and ORTEP plots

Form C

REFNR01_ALERT_3_A Ratio of reflections to parameters is < 6 for a
centrosymmetric structure
sine(theta)/lambda 0.5003
Proportion of unique data used 1.0000
Ratio reflections to parameters 5.2963
THETM01_ALERT_3_A The value of sine(theta_max)/wavelength is less than 0.550
Calculated sin(theta_max)/wavelength = 0.5003
PLAT027_ALERT_3_A _diffrn_reflns_theta_full (too) Low .... 15.00 Deg.
PLAT029_ALERT_3_A _diffrn_measured_fraction_theta_full Low 0.644
PLAT088_ALERT_3_C Poor Data / Parameter Ratio 9.59
PLAT220_ALERT_2_C Large Non-Solvent C Ueq(max)/Ueq(min) ... 3.6 Ratio
PLAT222_ALERT_3_C Large Non-Solvent H Uiso(max)/Uiso(min) .. 4.2 Ratio
PLAT230_ALERT_2_C Hirshfeld Test Diff for S1 -- C9 .. 5.5 su
PLAT234_ALERT_4_C Large Hirshfeld Difference O3 -- C8 .. 0.16 Ang.

PLAT241_ALERT_2_C Check High Ueq as Compared to Neighbors for C19

PLAT242_ALERT_2_C Check Low Ueq as Compared to Neighbors for C7

PLAT340_ALERT_3_C Low Bond Precision on C-C Bonds ....0.0098 Ang.

High-pressure data, these alerts are not unusual and are to be ascribed to the physical restrictions imposed by the pressure cell and limited access to reciprocal space. Data to parameter ratio was actually increased to 6.1 with the use of restraints. Data collected from the crystal in two orientations were merged together.

PLAT157_ALERT_4_C Non-standard Monoclinic Beta Angle less 90 Deg 88.21 Deg.

The non-standard setting was chosen to facilitate comparison with the structure predicted by theory.

PLAT201_ALERT_2_B Isotropic non-H Atoms in Main Residue(s) 4

These correspond to disordered propyl and 3-pentyl side chains.

PLAT031_ALERT_4_B Refined Extinction Parameter within Range ....2.333 Sigma

The four strongest reflections show a systematic trend of Fo < Fc. In view of this and of the crystal growth conditions we believe refinement of an extinction parameter is meaningful.

PLAT414_ALERT_2_C Short Intra D-H..H-X H4 .. H21A .. 1.98 Ang.

Noted, no action taken. These distances are not exceedingly short. Some of these atoms are disordered; it is possible that this distance is an artifact of refinement. H-atoms were placed in calculated positions.

Form A at 295 K

PLAT019_ALERT_1_B Check _diffrn_measured_fraction_theta_full/_max 0.945

THETM01_ALERT_3_C The value of sine(theta_max)/wavelength is less than 0.590

Calculated sin(theta_max)/wavelength = 0.5883

The required data resolution has been met. This is ambient-temperature data and the structure is disordered. Data are 99% complete to Theta(max)= 24.718

PLAT220_ALERT_2_B Large Non-Solvent C Ueq(max)/Ueq(min) ... 5.4 Ratio

PLAT242_ALERT_2_B Low Ueq as Compared to Neighbors for ..... C7 Check

PLAT242_ALERT_2_C Low Ueq as Compared to Neighbors for ..... C8 Check

PLAT241_ALERT_2_C High Ueq as Compared to Neighbors for ..... C19 Check

PLAT222_ALERT_3_C Large Non-Solvent H Uiso(max)/Uiso(min) .. 6.3 Ratio

PLAT230_ALERT_2_C Hirshfeld Test Diff for C5 -- C7 .. 7.0 su

PLAT230_ALERT_2_C Hirshfeld Test Diff for C16 -- C21 .. 5.5 su

PLAT234_ALERT_4_C Large Hirshfeld Difference C24A -- C25A .. 0.20 Ang.

Disordered structure.

PLAT157_ALERT_4_C Non-standard Monoclinic Beta Angle less 90 Deg 88.74 Deg.

This angle has been chosen to allow direct structural comparison with the low-temperature polymorph.

PLAT340_ALERT_3_C Low Bond Precision on C-C Bonds .... 0.0045 Ang.

PLAT413_ALERT_2_C Short Inter XH3 .. XHn H6B .. H25A .. 2.08 Ang.

PLAT414_ALERT_2_C Short Intra D-H..H-X H4 .. H21A .. 1.97 Ang.

Noted, no action taken. These distances are not exceedingly short. Some of these atoms are disordered; it is possible that this distance is an artifact of refinement. H-atoms were placed in calculated positions.

PLAT906_ALERT_3_C Large K value in the Analysis of Variance ...... 2.313 Check

PLAT911_ALERT_3_C Missing # FCF Refl Between THmin & STh/L= 0.588 40 Why ?

Noted, no action taken. Large K value for the lowest resolution shell might arise from data integration, scaling or absorption correction.

Form A at 223 K

THETM01_ALERT_3_A The value of sine(theta_max)/wavelength is less than 0.550

Calculated sin(theta_max)/wavelength = 0.5043

PLAT019_ALERT_1_B Check _diffrn_measured_fraction_theta_full/_max 0.591

REFNR01_ALERT_3_C Ratio of reflections to parameters is < 10 for a

centrosymmetric structure

sine(theta)/lambda 0.5043

Proportion of unique data used 1.0000

Ratio reflections to parameters 8.5071

PLAT088_ALERT_3_C Poor Data / Parameter Ratio 8.61

PLAT148_ALERT_3_C su on the a - Axis is (Too) Large .... 0.015 Ang.

PLAT148_ALERT_3_C su on the b - Axis is (Too) Large .... 0.0130 Ang.

PLAT148_ALERT_3_C su on the c - Axis is (Too) Large .... 0.030 Ang.

PLAT340_ALERT_3_C Low Bond Precision on C-C Bonds .... 0.0060 Ang.

Upon cooling and during the data collection the crystal splits into several fragments. Reflections get progressively broader with more domains building into the diffraction. The structure presented here comes from one major domain that survives for three of the five runs collected, with satisfactory crystal centring. Several cooling experiments were attempted in order to improve data quality - the data presented here is the best we could do using a home source.

PLAT220_ALERT_2_B Large Non-Solvent C Ueq(max)/Ueq(min) ... 5.5 Ratio

PLAT222_ALERT_3_C Large Non-Solvent H Uiso(max)/Uiso(min) .. 6.3 Ratio

PLAT242_ALERT_2_C Low Ueq as Compared to Neighbors for ..... C7 Check

PLAT242_ALERT_2_C Low Ueq as Compared to Neighbors for ..... C8 Check

The structure is disordered. Please also see comments above.

PLAT157_ALERT_4_C Non-standard Monoclinic Beta Angle less 90 Deg 88.54 Deg.

This angle has been chosen to allow direct structural comparison with the low-temperature polymorph.

PLAT413_ALERT_2_B Short Inter XH3 .. XHn H6B .. H25A .. 1.91 Ang.

PLAT413_ALERT_2_C Short Inter XH3 .. XHn H19B .. H27D .. 2.14 Ang.

PLAT414_ALERT_2_C Short Intra D-H..H-X H4 .. H21A .. 1.98 Ang.

Noted, no action taken. These distances are not exceedingly short. Some of these atoms are disordered; it is possible that this distance is an artifact of refinement. H-atoms were placed in calculated positions.

Form B

THETM01_ALERT_3_A The value of sine(theta_max)/wavelength is less than 0.550

Calculated sin(theta_max)/wavelength = 0.5498

The IUCr sin(theta)/lambda requirement of 0.6 has actually been reached. Copper radiation used.

PLAT222_ALERT_3_C Large Non-Solvent H Uiso(max)/Uiso(min) ..5.4 Ratio

PLAT245_ALERT_2_C U(iso) H4 Smaller than U(eq) N4 by ... 0.013 AngSq

Noted, no action taken.

Supplementary References

1. Kresse, G. & Hafner, J. Ab initio molecular dynamics for liquid metals. *Phys. Rev. B* **47,** 558-561 (1993).

2. Kresse, G. & Furthmüller, J. Efficiency of ab-initio total energy calcualtions for metals and semiconducturs using a plane-wave basis set. *Comput. Mat. Sci.* **6,** 15-50 (1996).

3. Kresse, G. & Furthmüller, J. Efficient iterative schemes for ab initio total-energy calculations using a plane-wave basis set, *Phys. Rev. B* **54,** 11169-11186 (1996).

4. Kresse, G. & Joubert, D. From ultrasoft pseudopotentials to the projector augmented-wave method. *Phy. Rev. B* **59,** 1758-1775 (1999).

5. Grimme, S., Antony, J., Ehrlich, S. & Krieg, H. A consistent and accurate ab initio parametrization of density functional dispersion correction (DFT-D) for the 94 elements H-Pu. *J. Chem. Phys.* **132,** 154104-154119 (2010).

6. Neumann, M. A. & Perrin, M.-A. Energy ranking of molecular crystals using density functional theory calculations and an empirical van der Waals correction. *J. Phys. Chem. B* **109,** 15531-15541 (2005).

7. Frenkel, D. & Smit, B. *Understanding Molecular Simulation*. (Academic Press, San Diego, 2002).

8. Moggach, S. A., Allan, D. R., Parsons, S. & Warren, J. E. Incorporation of a new design of backing seat and anvil in a Merrill–Bassett diamond anvil cell. *J. Appl. Cryst.* **41,** 249-251 (2008).

9. Piermarini, G. J., Block, S., Barnett, J. D. & Forman, R. A.Calibration of the Pressure Dependence of the R1 Ruby Fluorescence Line to 195 kbar. *J. Appl. Phys.* **46,** 2774-2780 (1975).

10. Dawson, A., Allan, D. R., Parsons, S. & Ruf, M. Use of a CCD diffractometer in crystal structure determinations at high pressure. *J. Appl. Cryst.* **37,** 410-416 (2004).

11. Altomare, A., Cascarano, G., Giacovazzo, C. & Guagliardi, A. Completion and refinement of crystal structures with SIR92. *J. Appl. Cryst.* **26,** 343-250 (1993).

12. Sheldrick, G. M. A short history of SHELX. *Acta Crystallogr.* **A64,** 112-122 (2008).

13. Hübschle, C. B., Sheldrick, G. M. & Dittrich, B. A Qt graphical user interface for SHELXL. *J. Appl. Cryst.* **44,** 1281-1284 (2011).

14. Thorn, A., Dittrich, B. & Sheldrick, G. M. Enhanced rigid-body restraints. *Acta Crystallogr.* **A68,** 448-451 (2012).

15. Watkin, D. J. Structure refinement: some background theory and practical strategies. *J. Appl. Cryst.* **41,** 491-522 (2008).

16. Bürgi, H. B. Motion and disorder in crystal structures analysis: Measuring and distinguishing them. *Annu. Rev. Phys. Chem.* **51,** 275-296 (2000).

17. Bond, A. D., Boese, R. & Desiraju, G. R. On the polymorphism of aspirin: crystalline aspirin as intergrowths of two “polymorphic” domains. *Angew. Chem. Int. Ed.* **46,** 618-622 (2007).
